# Multifaceted DNA metabarcoding of guano to uncover multiple classes of ecological data in two different bat communities

**DOI:** 10.1111/eva.13425

**Published:** 2022-06-29

**Authors:** Richard F. Lance, Xin Guan, Joel F. Swift, Christine E. Edwards, Denise L. Lindsay, Eric R. Britzke

**Affiliations:** ^1^ Environmental Laboratory US Army Engineer Research & Development Center Vicksburg Mississippi USA; ^2^ Bennett Aerospace Vicksburg Mississippi USA; ^3^ Center for Conservation and Sustainable Development, Missouri Botanical Garden St. Louis Missouri USA; ^4^ Moderna, Inc. Cambridge Massachusetts USA; ^5^ Department of Biology St. Louis University St. Louis Missouri USA

**Keywords:** Chiroptera, DNA barcode, DNA sexing, noninvasive genetics, trophic analysis, wildlife disease surveillance

## Abstract

DNA contained in animal scat provides a wealth of information about the animal, and DNA metabarcoding of scat collections can provide key information about animal populations and communities. Next‐generation DNA sequencing technologies and DNA metabarcoding provide an efficient means for obtaining information available in scat samples. We used multifaceted DNA metabarcoding (MDM) of noninvasively collected bat guano pellets from a *Myotis lucifugus* colony on Fort Drum Military Installation, New York, USA, and from two mixed‐species bat roosts on Fort Huachuca Military Installation, Arizona, USA, to identify attributes such as bat species composition, sex ratios, diet, and the presence of pathogens and parasites. We successfully identified bat species for nearly 98% of samples from Fort Drum and 90% of samples from Fort Huachuca, and identified the sex for 84% and 67% of samples from these same locations, respectively. Species and sex identification matched expectations based on prior censuses of bat populations utilizing those roosts, though samples from some species were more or less common than anticipated within Fort Huachuca roosts. Nearly 62% of guano samples from Fort Drum contained DNA from *Pseudogymnoascus destructans*, where bats with wing damage from White‐nose Syndrome were commonly observed. Putative dietary items were detected in a majority of samples from insectivorous bats on Fort Drum (81%) and Fort Huachuca (63%). A minority of guano samples identified as the nectarivorous *Leptonycteris yerbabuenae* (28%) provided DNA sequences from putative forage plant species. Finally, DNA sequences from both putative ecto‐ and endoparasite taxa were detected in 35% and 56% of samples from Fort Drum and Fort Huachuca, respectively. This study demonstrates that the combination of noninvasive sampling, DNA metabarcoding, and sample and locus multiplexing provide a wide array of data that are otherwise difficult to obtain.

## 1 INTRODUCTION

Ecologists have been afforded unprecedented access to information contained in animal scat via a combination of technological advances in DNA sequencing and associated growth in DNA sequence databases (i.e., molecular scatology; Bohmann et al., 2018; Lopes et al., 2020; Ratnasingham & Hebert, 2007; Reed et al., 1997; Swift et al., 2018). Multifaceted DNA metabarcoding (MDM; Swift et al., 2018) of guano samples is an example of an advanced molecular scatology approach, and involves high‐throughput sequencing of DNA libraries enriched for DNA barcode and other diagnostic loci targeting a variety of different data classes. Swift et al. (2018) recently showed MDM to be an efficient and accurate method, capable of providing descriptive data to include bat species identification, sex, diet, pathogens, and parasites. High‐throughput molecular DNA diagnostic applications, like MDM, that are based on noninvasively collected samples have several qualities that make them highly desirable for studies of wildlife. For one, collection of noninvasive samples does not require handling or otherwise causing stress to study organisms. Two, noninvasive sample collection does not require specialized training. Three, scat may contain DNA evidence for many different key organismal attributes or states, which can be uncovered using different “universal” assays (Guan et al., 2020, 2021; Swift et al., 2018; Walker et al., 2016; Zeale et al., 2011). Further, some of these attributes, such as the presence of endoparasites and diet, are very difficult to quantify using traditional approaches (involving detailed necropsies or microscopic analyses of scat), and the use of DNA metabarcoding may vastly improve the ease and efficiency of collecting these data (Edwards et al., 2019; Swift et al., 2018). Finally, if stored properly, DNA extracts from these samples may remain viable for many years, allowing for future use including recharacterization using more advanced assays or for capturing later‐emerging data of interest.

In this study, we utilized MDM to understand a range of attributes of a colony of *Myotis lucifugus* Le Conte (Little Brown Bat) in the northeastern United States of America (USA) and mixed‐species roosts in the southwestern USA. The goal of this study was to use noninvasively collected bat fecal samples, to use MDM to provide information on bat species, sex, diet, and endo‐ and ectoparasites for each sample. In the case of the *M. lucifugus* colony, we also incorporated an assay for the presence of DNA of the fungal pathogen *Pseudogymnoascus destructans* (*Pd*) that is responsible for White‐nose Syndrome (WNS) in bats.

## 2 MATERIALS AND METHODS

### 2.1 Study systems and guano collection

The first study system was a single‐species maternity colony of *M. lucifugus* located in a constructed bat house on the US Army Installation at Fort Drum, NY, USA (Fort Drum). This species is found throughout much of the Nearctic region and was once among the most commonly encountered bats throughout the northeastern United States and eastern Canada (Fenton, 1980; Frick et al., 2010). However, following the emergence of WNS, a disease resulting from infection of bat tissues by the fungal pathogen *Pd*, populations of many bats of the eastern United States and Canada, including *M. lucifugus*, have declined precipitously (Frick et al., 2010; Hoyt et al., 2021). The second study system was a combination of roosts on the US Army Installation at Fort Huachuca, AZ, USA (Fort Huachuca). One site is a cave that houses day‐roosting maternity colonies of the nectarivorous *Leptonycteris yerbabuenae* Martínez and Villa‐R (Lesser Long‐nosed Bat) and the insectivorous *Myotis velifer* Allen (Cave Myotis) (Sidner & Stone, 2003), as well as small night‐roosting groups of the primarily insectivorous *Antrozous pallidus* Allen (Pallid Bat). The other site, a concrete bridge located approximately 6 km from the cave, is used as a night‐roost by, among other bat species, *L. yerbabuenae*, *M. velifer*, and *A. pallidus* (E. Britzke & R. Lance, personal observation).

Guano was collected on Fort Drum by placing disposable plastic sheeting underneath the bat house in late May 2016. Sheeting was left out over the course of 3 days and guano samples were collected from the sheeting each morning. During the collection period, the bat house was known to contain about 120 *M. lucifugus*. We used sterile, single‐use tweezers to place each guano pellet into an individual 2.0 ml screw‐cap tube containing silica gel desiccant. Samples were stored at room temperature in cardboard boxes to reduce potential light‐induced DNA degradation.

**FIGURE 1:**
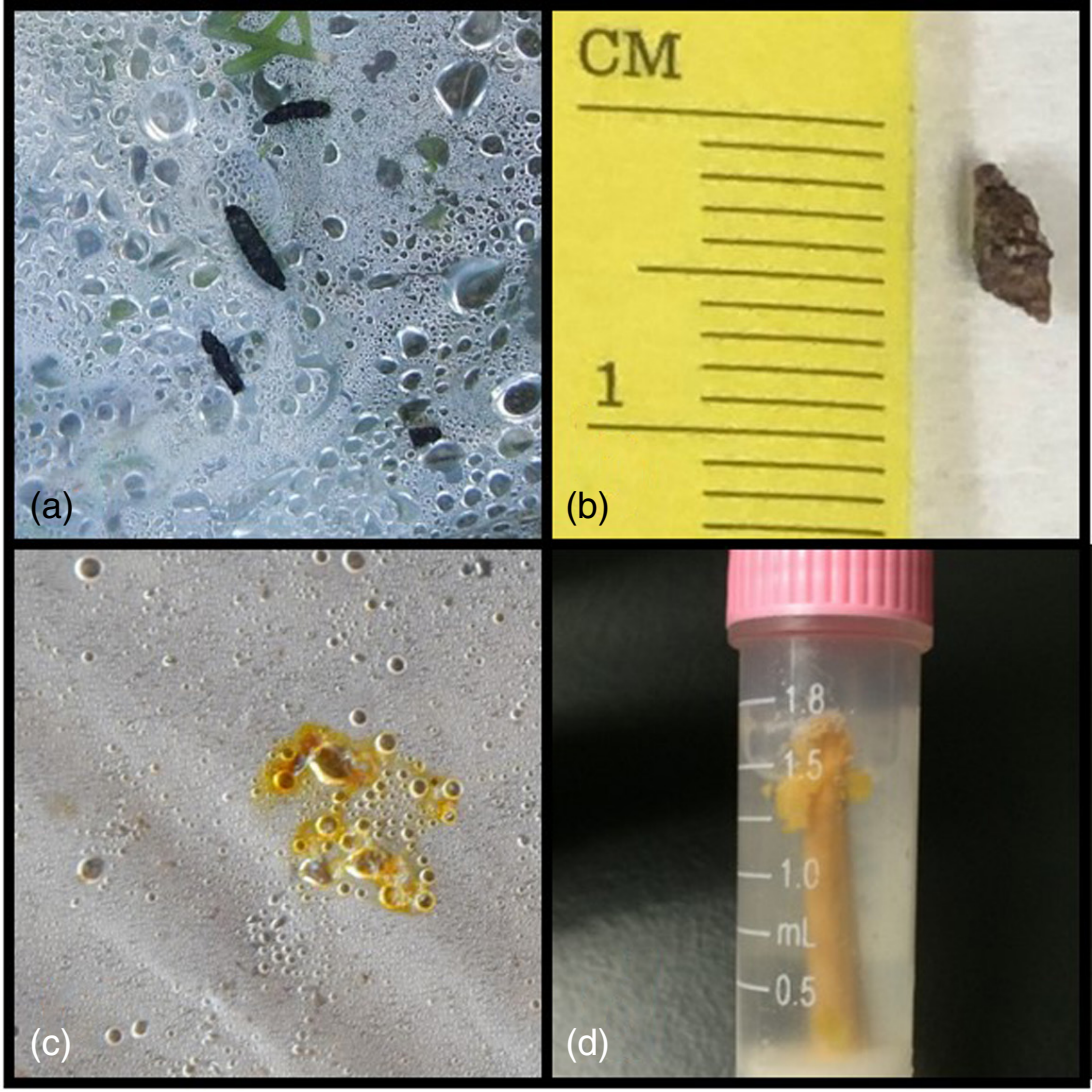
Guano samples from insectivorous (a, b) and nectarivorous (c, d) bats, as collected in the field on tarps (a, c) and stored in tubes with desiccant (b) and RNAlater (d). The general size scale of guano samples is demonstrated in panel b

On Fort Huachuca, guano samples were collected in September 2016. Disposable plastic sheeting was placed on the floor of the outer chamber of the cave, as well as underneath the bridge roost. During the collection period, circa 25,000 *L. yerbabuenae* and a smaller maternity colony (circa 5000) of *M. velifer* utilized the cave as a day roost, and *A. pallidus* were known to utilize the cave entrance chamber as a night roost. The bridge roost is used primarily by *L. yerbabuenae*, *M. velifer*, and *A. pallidus*. At both locations, bat scat generally came in two forms: solid guano pellets and “splats,” or liquid stool produced by nectarivorous bats (Figure 1). Pellets were collected as described above. Splats, which had generally dried before collection, were scraped into 2.0 ml tubes containing RNA later (Sigma‐Aldrich) preservative using sterile disposable wooden sticks.

### 2.2 DNA extraction, library preparation, and sequencing

DNA sample processing and analysis closely followed methods described previously (Swift et al., 2018). The DNA extractions and initial PCR steps included no‐template controls (NTC). DNA was extracted from guano pellets and splat samples using a CTAB protocol (cetrimonium bromide; Doyle & Doyle, 1987), which was modified with smaller lysis and wash volumes, and an added 95% ethanol wash. DNA extraction from guano samples, polymerase chain reactions (PCRs), and post‐PCR processing (e.g., next‐generation sequencing) were conducted in separate rooms that did not share airflow. As a means to ascertain the general quality of sample DNA extracts, DNA concentration and purity (260/280 nm wavelength ratio) were measured for a subset of samples using a Nanodrop™ 1000 spectrophotometer (Thermo Fisher Scientific).

Each sample (and NTC) next underwent separate PCR enrichments (i.e., PCR amplifications) for target loci (the DNA barcodes and diagnostic markers used to identify bat species and sex, and to detect the presence of *Pd* DNA, the DNA of potential arthropod and/or plant dietary items, and the DNA of potential parasites). The PCR primers employed for these assays are listed in Table 1. All PCRs were prepared within a sterile laminar flow hood; hood surfaces were sterilized with a 10% bleach solution and then treated with ultraviolet light for 15 min prior to PCR preparation. The enrichment PCRs and library preparation for sequencing followed the Illumina® *16S* metagenomic protocol (Illumina, 2013), with some modifications (detailed below), and involved two rounds of PCR, each followed by a cleanup step. In the first stage, independent PCRs were run for each combination of sample and locus. All primers utilized for this first‐stage PCR (Table 1) also incorporated a 5′ Nextera overhang sequence (Illumina, Inc.). These PCRs were conducted in 25 μl reactions containing 12.5 μl of 2 × KAPA HotStart ReadyMix (Roche), 1 μl of DNA, 4 pmol of each forward or reverse primer, and Ambion® nuclease‐free water (Invitrogen™) to the final concentration. PCR temperature cycling conditions were as follows: (i) 3 min at 94°C, (ii) denaturation for 30 s at 94°C, (iii) annealing for 30 s at 52°C, (iv) extension for 45 s at 72°C, (v) 34 repetitions of steps ii–iv, and (vi) a final elongation at 72°C for 10 min. Amplicons from each sample (and NTC) and locus combination were then cleaned and length‐filtered using AMPure XP beads (Beckman Coulter) following the manufacturer's standard protocol and a bead:DNA ratio of 1:1.6. Amplicon concentrations were measured with a Qubit v.2 fluorometer and a dsDNA HS assay kit (Thermo Fisher Scientific). Amplicons from the first stage PCR (i.e., different loci for each sample or NTC) were pooled at the sample level to equimolar concentrations (110 ng/μl) and then amplified using the Nextera Index Kit (Illumina, Inc.) to incorporate sample‐specific multiplex identifier indices and sequencing adapters. PCRs contained 25 μl of 2 × KAPA HotStart ReadyMix, 5 μl of the sample amplicon pool, 5 μl of each of two indices from the Nextera Index Kit, and 10 μl Ambion® nuclease‐free water. PCR temperature cycling conditions were as follows: (i) 3 min at 95°C, (ii) denaturation for 30 s at 95°C, (iii) annealing for 30 s at 55°C, (iv) extension for 30 s at 72°C, (v) eight repetitions of steps ii–iv, and (vi) a final elongation at 72°C for 5 min. The amplicons from this second round of PCRs were then cleaned, length‐filtered, and quantified as described above. Equal volumes of indexed amplicons for each sample were then pooled as a sample library and normalized to a concentration of 4 nM using resuspension buffer (Illumina, 2020). Each sample library was then denatured and diluted to a final concentration of 8–10 pM and combined with PhiX control (to a PhiX concentration of 10%) according to Illumina guidelines (Illumina, 2017). Finally, the pooled sets of 94 sample libraries, along with two PCR NTCs, were sequenced on an Illumina® MiSeq using the MiSeq Reagent Kit v3 (600‐cycle; Illumina, Inc.). Metadata for the MDM sequencing runs can be found in Table S1.

**TABLE 1:** Primer pairs used to target select taxonomic groups, with targeted DNA loci, expected ranges of amplicon sizes, and key citation describing primers

Targeted data class	PCR primers	DNA locus	Expected amplicon length	Citation
Bat species	Ins16S_1_F, Ins16S_1_R	*16SrDNA*	286–292	Clarke et al. (2014)
Bat sex	XGXC‐F, XGXC‐R	*Zinc Finger X*	175	Swift et al. (2018)
	XGYC‐F, XGYC‐R	*Zinc Finger Y*	120	Guan et al. (2020)
Bat sex^H^	XGXC‐F.ly, XGXC‐R.ly	*Zinc Finger X*	250	
	XGYC‐F.ly, XGYC‐R.ly	*Zinc Finger Y*	190	
Bat sex^H^	KXZF‐F, KXZF‐R	*Zinc Finger X*	245	Korstian et al. (2013)
	KYZF‐F, KYZF‐R	*Zinc Finger Y*	80	
*Pd* ^D^	Nu‐IGS‐0169‐5′, Nu‐IGS‐0235‐3′	*IGS*	103	Muller et al. (2013)
Arthropod diet	Ins16_1_F, Ins16_1_R	*16SrRNA*	191–261	Clarke et al. (2014)
Plant diet^H^	trnHR2, psbAF	*trnH‐psbA*	185–887	Sang et al. (1997)
Bat endoparasites	MN18F, 22R reverse	*18SrDNA*	345	Bhadury et al. (2006)

*Note*: Expected amplicon size ranges are approximate and include forward and reverse primers. *Pd* refers to *Pseudogymnoascus destructans*, the fungal species associated with White‐nose Syndrome in bats. ^H^Designates an assay performed solely for samples from Fort Huachuca, AZ. ^D^Designates an assay performed solely for samples from Fort Drum, NY. All other assays were performed for both sample sets.

### 2.3 Sequence processing and analysis

Sequence reads were demultiplexed to individual sample datasets using Illumina's CASAVA v1.8 software. We then employed a custom shell script, which we termed the multifaceted DNA metabarcoding (MDM) pipeline (Swift et al., 2018; https://github.com/Kenizzer/Bat_MDM), to further process reads through merging of paired reads with quality control, demultiplexing by locus‐specific primers, amplicon‐size and depth filtering, and BLAST (Basic Local Alignment Search Tool; Altschul et al., 1990) for each remaining amplicon sequence variant (ASV). MDM incorporated several functions from OBITOOLS v1.01 (Boyer et al., 2016). All sequence data processing and analysis were conducted on a multicore desktop and, where possible, GNU Parallel (Tange, 2018) was used to parallelize tasks. As part of initial read processing, consensus sequences of paired reads with alignment scores greater or equal to 40 were kept, whereas paired sequences with scores less than 40 were concatenated (illuminapairedend tool, “score‐min” option; Boyer et al., 2016). For all data classes, only ASVs close to the expected amplicon size ranges (Table 1) and with read counts 2–3× greater than those found for ASVs in NTCs were reserved for further analysis (NTC results found in Table S2). Only BLAST results with ≥98% query sequence coverage were retained. The specific steps employed to process each data class are specified in Section 2.4 below.

### 2.4 ASV classification for each data class

#### 2.4.1 Bat species classification

For each sample, ASVs resulting from the *16S* ribosomal DNA (rDNA) barcode (Table 1) enrichment were imported into Geneious 2019.0 (Biomatters, Ltd.) and BLAST searched against a custom sequence database containing all available Microchiroptera (Mammalia:Chiroptera) *16S rRNA* DNA sequence data found in the GenBank Nucleotide Database on Dec 31, 2018. ASVs were only retained if they fell within the approximate expected size range (240–260 bp), exhibited read counts above the NTC threshold, and if BLAST results provided a ≥95% sequence match to at least one of the reference bat *16S* sequences. Retained ASVS were further BLAST searched against the entire GenBank Nucleotide Database (January 2019) in order to further verify the BLAST result from the custom database.

#### 2.4.2 Bat sex classification

Amplicon sequence variants from the different sex chromosome locus enrichments (Table 1) for each sample were imported into Geneious and BLAST searched against a custom database containing all *ZF* sequences for Microchiroptera in GenBank (downloaded 09 Jan 2019). Based on observations on patterns of sequence matching, only BLAST results with sequence matches with *e*‐values ≤ 1e^−20^ were retained. Retained ASVS were then further BLAST searched against the entire GenBank Nucleotide Database (November 2020) in order to further verify that the best available sequence match for the ASV was a bat sex chromosome. Because there were no clear trends in the ratio of X‐chromosome: Y‐chromosome read counts, for either set of sex markers, any sample with Y‐chromosome ASVs (above the NTC read count threshold) was assigned as male.

#### 2.4.3 White‐nose causative agent

Amplicon sequence variants from the *Pd* enrichment (Table 1) were imported into Geneious and BLAST searched against the expected amplicon sequence (e.g., GenBank Accession JX270192.1; Lorch et al., 2013). Based on sequence identity patterns in *Pd* sequences archived in the NCBI database, only ASVs with BLAST sequence match ≥98.5% sequence match were retained. WNS has not yet been observed in Arizona or surrounding states, so this assay was not employed for samples from Fort Huachuca. No *Pd* ASVs were observed in the NTCs.

#### 2.4.4 Diet characterization

Amplicon sequence variants from the *16S rRNA* marker (Table 1) that were in the size range of 85–325 bp (and at read counts above the NTC threshold) were BLAST searched against the GenBank Nucleotide database (02 Feb 2019). Those ASVs with match *e*‐values ≤ 1e^−30^ to sequences in the database were retained. Diet characterization for nectivorous bats, using a *trnH‐psbA locus* (Table 1), was conducted for Fort Huachuca samples only. Here, ASVs greater than 85 bp were searched against the GenBank Nucleotide database (Feb 2, 2019). ASVs with sequence match *e*‐values ≤ 1e^−30^ to GenBank sequences were retained and imported into MEGAN 6.14.13 (Huson et al., 2016; settings described in Figure S1) in order to review and summarize taxonomic classification.

#### 2.4.5 Parasite characterization

For parasite characterization in all samples, *18S* RNA ASVs were processed and filtered as described above for *16S* RNA ASVs, with the exception that only ASVs in the size range of 240–350 bp (and at read counts above the NTC threshold) were retained.

## 3 RESULTS

### 3.1 Sampling

A total of 376 guano samples were collected from under the artificial roost structure on Fort Drum, and a total 274 guano samples were obtained from Fort Huachuca, including 102 from the cave roost and 172 from the bridge roost. The Fort Huachuca guano samples included 26 and 31 nectar‐feeding bat splats (as determined by scat structure; Figure 1) from the cave and bridge roosts, respectively.

### 3.2 Classification of samples to bat species

Nearly all of the Fort Drum samples (368 of 376 samples, 97.9%) contained reads from the ins16S_1 assay (*16S rRNA*; Table 1) that were within the expected size range and had read counts above the cut‐off value. Of these, 366 were assigned to *M. lucifugus*, all with sequence matches of at least 99.6% to *M. lucifugus* 16S rRNA sequence. Two ASVs were assigned to *Eptesicus fuscus* Palisot de Beauvois (Big Brown Bat), both with sequence matches of 100% to *E. fuscus* 16S rRNA sequence. Although *M. lucifugus* sequences for this locus maybe identical or highly similar to homologous sequence in some other *Myotis* species (Guan et al., 2020), no such species occurs in the sampled region.

For Fort Huachuca, 245 of 274 guano samples were assigned to a bat species (89.4%; Table 2), five samples exhibited similar read count totals for more than one species (“mixed”), indicating cross‐contamination of samples or other errors (e.g., index hopping during sequencing), and 22 samples failed to provide bat ASVs with read counts above the cut‐off value. The *16S* locus used for identification is identical for *M. velifer* and *M*. *yumanensis* and thus cannot differentiate between the two species. The *16S* locus also cannot differentiate *M. evotis* Allen (Long‐eared Myotis), *M. occultus* Hollister (Arizona Myotis), *M. lucifugus*, and *M. thysanodes*; however, because only *M. thysanodes* occurs in the study area, the ASVs from the sequence group were assigned to this species.

**TABLE 2:** Bat species assignments for guano samples taken from two sites on Fort Huachuca, AZ, with sample numbers and sequence match percentages for amplicon sequence variants associated with each detected species

Fort Huachuca	*Antrozous pallidus*	*Leptonycteris yerbabuenae*	*Myotis velifer* or *Myotis yumanensis*	*Myotis thysanodes*	Unknown or mixed
Cave	50	40	0	0	12
Bridge	34	20	99	2	15
% Sequence match	99.6%	100%	99.6%–100%	100%	

### 3.3 Bat sex identification

Of the 366 Fort Drum samples identified as *M. lucifugus*, we identified sex from 316 samples (86.3%), all of which were identified as female (100%). The two *E. fuscus* samples were also assigned as female. Samples from which sex could not be assigned contained either read counts for the sex chromosomes below the cut‐off derived from no‐template controls or no sex chromosome reads.

For Fort Huachuca, sex chromosome read counts were generally much weaker than those obtained from Fort Drum. In terms of the proportion of samples for which we were able to obtain sex chromosome data, the XGXC, XGYC, XGXC.ly, and XGYC.ly primer sets performed better than the KXZF and KYZF primer sets, and only the former were used to assign sex to each sample. Of the 245 Fort Huachuca samples assigned to species, sex identification was possible for 163 (67%; Table 2). Sex assignment was particularly poor for *A. pallidus* and *L. yerbabuenae* samples collected at the night‐roost (3% and 17% assigned, respectively; Table 2), despite much higher assignment rates for samples from the same species collected from the cave roost (74% and 86%, respectively), and much higher assignment rates for putative *M. velifer* samples from the bridge roost (90%).

### 3.4 Determination of *Pd* exposure

Of the 376 samples from Fort Drum, 229 (62.4%) contained *Pd* DNA. No *Pd* ASVs were observed in no‐template controls. 99% of *Pd* positive samples shared a single ASV haplotype with a 100% match to GenBank *Pd* accession number JX415267 (Muller et al., 2013).

### 3.5 Bat diets

For the 376 Fort Drum guano samples, *16S rRNA* ASVs that were likely derived from bat prey were obtained from 303 samples (80.6%), while 40 samples (10.6%) contained such ASVs from the *18S rRNA* dataset. Between the two barcode loci, prey ASVs were detected in a total of 305 samples (81.1%). All prey items in the final dataset were assigned to Class Insecta or Arachnida, with DNA from Orders Diperta, Trichoptera, and Ephemeroptera each appearing in numerous samples (*N* > 150 per order). ASVs from orders Coleoptera, Lepidoptera, and Aranae were encountered in fewer, though still substantial numbers of samples (*N* = 24–149), whereas DNAs from other orders, including Hemiptera, Hymenoptera, Mecoptera, Megaloptera, Neuroptera, Odonata, Orthroptera, Plecoptera, Psocoptera, Thysanoptera, and Opiliones were found in only a few samples (*N* < 25). Further details on classifications to family, genus, and species for guano ASVs for both bat species detected in the Fort Drum dataset are found in Table S3.1a,b.

For the 245 Fort Huachuca guano samples, 70 (28.6%) contained *16S rRNA* ASVs from likely prey items, including 68 of the 117 (58.1%) samples from insectivorous bats. For the *18S rRNA* locus, 100 samples (40.8%) also provided some ASVs that likely corresponded to bat prey. All ASVs in this dataset were assigned to either Class Insecta, Class Arachnida, or Class Chilopoda. For *M. velifer*/*yumanensis*, ASVs from Order Coleoptera were encountered most frequently (*N* = 41 samples), with smaller numbers of samples (*N* = 1–6) containing ASVS from the orders Blattodea, Diptera, Hymenoptera, Lepidoptera, Neuroptera, Psocoptera, and Aranae. For *A. pallidus*, ASVs from Order Orthroptera and Coleoptera were encountered most frequently (*N* = 57 and 10 samples, respectively), with smaller numbers of samples (*N* = 1–5) containing ASVS from the orders Diptera, Hemiptera, Hymenoptera, Lepidoptera, Mantodea, Neuroptera, Phasmatodea, Psocoptera, and Scolopendromorpha. The two samples assigned to *M. thysanodes* both provided a single ASV classified to Lepidoptera (Insecta). Seven *L. yerbabuenae* samples provided ASVs that could be assigned to non‐parasite arthropods, including the arachnid order Araneae and the insect orders, Lepidoptera, Orthroptera, and Thysanoptera. Between the two barcode loci, likely prey ASVs were detected in a total of 124 samples (48.7%). Further details on classifications to family, genus, and species for ASVs from guano samples from all bat species are found in Table S3.2a–d.

A total of 29 of the 245 guano samples from Fort Huachuca (11.8%) provided *trnH‐psbA* ASVs classified to Kingdom Plantae. Of these samples, only three were determined to come from the primarily nectarivorous *L. yerbabuenae* (1 splat and 2 guano pellets; 5% of all *L. yerbabuenae* samples). Plant ASVs were also obtained from the *18S* barcode data for 85 of the 245 guano samples (34.7%), including 16 samples from *L. yerbabuenae* (8 splat and 8 guano pellets; 26.7% of all *L. yerbabuenae* samples). Between these two barcode loci, plant ASVs were detected in a total of 100 samples (40.8%), and 17 of the 60 *L. yerbabuenae* samples (28.3%). In the *L. yerbabuenae* samples, plant ASVS were classified to nine orders within Class Magnoliopsida, including several samples with ASVs from Asparagales (*N* = 10) or Myrtales (*N* = 6), along with two or fewer samples containing ASVs from Asterales, Commelinales, Fabales, Gentianales, Lamiales, Poales, and Rosales. Further details on plant ASV classifications for *L. yerbabuenae*, *A. pallidus*, and *M. velifer*/*M. yumanensis* samples are found in Table S3.3a–c.

### 3.6 Bat parasites

For Fort Drum, *18S rRNA* ASVs that likely correspond to bat ectoparasites and endoparasites were obtained from 87 of the 376 guano samples (23.1%). All parasites were assigned to Phyla Apicomplexa (one order, one class), Arthropoda (two classes, three orders), Nematoda, or Platyhelminthes (two classes, two orders). *16S rRNA* ASVs that corresponded to likely bat parasites were detected in 52 samples (13.8%), including ASVs classified to the phyla Apicomplexa (one order, one class), Arthropoda (two classes, three orders), and Platyhelminthes (one class, one order). Between these two barcode loci, parasite ASVs were detected in a total of 130 samples (34.6%), with the Apicomplexan class Conoidasida (*N* = 78 samples), Arthropod class Arachnida (*N* = 26 samples), and Platyhelminth class Trematoda (*N* = 23 samples) being among the more common parasite ASVs encountered. Further details on classifications to family, genus, and species for ASVs from guano samples from both bat species are found in Table S4.1a,b.

For Fort Huachuca, *18S rRNA* ASVs that correspond to likely bat ectoparasites and endoparasites were obtained from 139 of the 245 guano samples (56.7%). All parasites were assigned to the phyla Apicomplexa (one class, one order), Arthropoda (two classes, four orders), Euglenozoa (one class, one order), Nematoda (one class, one order), and Platyhelminthes (one class, one order). The Apicomplexan class Conoidasida (*N* = 108 samples) was by far the most commonly encountered parasite ASV, with the Platyhelminth class Cestoda (*N* = 18), Arthropod classes Arachnida (*N* = 15 samples) and Insecta (*N* = 4), and Euglenozoan class Kinetoplastea (*N* = 9) also being encountered in several samples. Further details on classifications to family, genus, and species for ASVs from guano samples from these same bat species are found in Table S4.2a–d.

## 4 DISCUSSION

In this study, our objective was to understand the extent to which MDM and noninvasive sampling of bat guano could be used to uncover data on bat communities, including species, sex ratios, diet, and the presence of parasites and pathogens. We were able to arrive at species classifications for nearly all guano pellet samples collected from under a bat roost on Fort Drum, and from both guano pellets and nectar‐feeding bat splats under two roosts on Fort Huachuca. We were further able to classify bat sex for a substantial majority of those samples. For the Fort Drum samples, where WNS is prevalent, we detected the causal fungal agent *Pd* in a large number of samples. Dietary items and parasites were also discerned from DNA in the guano samples.

One common challenge for metabarcoding applications is the presence of low levels of false‐positive contamination, which can result from erroneous assignment of ASVs to samples due to tag‐jumping or index‐switching during sequencing, cross‐contamination among samples at some stage of sample procurement and processing, and/or contamination of samples with extrinsic DNAs in the field or lab (Drake et al., 2022; Sepulveda et al., 2020). Currently, there are no standard or “best” methods for accounting for such contamination in ASV filtering or analysis. In our study, we instituted a threshold for retaining ASVs based on comparison to read counts for the same ASV in our NTC sequencing runs. Retained ASVs were required to have read counts greater than at least 2–3× the read counts for that ASV in the NTCs. We shifted to the more conservative 3× threshold in cases where we observed a small number of samples with read counts for a particular ASV that were greater than 2× the read counts for the same ASVs in the NTCs, but much lower than corresponding read counts found in other samples; shifting to a 3× threshold eliminated these suspect observations. Our criterion for retaining ASVs was based on the rationale that a “zero‐tolerance” for taxa detected in the NTCs could result in loss of taxa that were common or at high concentrations in our samples, and thus critical data points (e.g., the bat species associated with the sample). Simply retaining an ASV found in a sample at a read count greater than that observed for the ASV in the NTCs (i.e., a “Max Contamination” filtering approach; Drake et al., 2022) would not take into account likely variation in sample contamination levels—here we treated the highest read count for an ASV in our NTCs as more of a central value for potential contaminant occurrence than as a maximum contamination level. The “Max Contamination” filtering approach has been found to be relatively effective for minimizing false positives, while minimizing the loss of true positives with low read counts (Drake et al., 2022), and our approach is a more conservative variation on this method. Additionally, in order to maximize the accuracy of taxon assignment, our ASV filtering approach was combined with amplicon size filtering and selective retention of identified taxa based on known geographic ranges and/or occurrence in records from past bat diet or parasite studies. We further note that we made no attempt at fine‐scale analyses comparing the diversities or numbers of ASVs (or operational taxonomic units; OTUs) detected in samples, which minimizes the influence of small (e.g., single nucleotide) PCR or sequencing errors on study outcomes.

Results from both study locales demonstrated key principles of using a noninvasive, scat‐based genetics approach like MDM for species identification at bat roosts. On Fort Drum, we collected individual guano pellet samples under a known *M. lucifugus* maternity roost and all but two samples were classified as *M. lucifugus*. The two exceptions were classified as *E. fuscus*, a common species in the area which has been observed to utilize this roost (C. Dobony, personal observation). Species identification from guano samples collected on Fort Huachuca was less straightforward. The cave day roost is utilized by tens of thousands of *L. yerbabuenae* and thousands of *M. velifer*, from which we classified 39% of samples to *L. yerbabuenae* and 49% to *A. pallidus* (12% of samples could not be identified to species), and no samples identified as *M. velifer*/*M. yumanensis*. The high frequency of samples from the cave roost identified to *A. pallidus* is not unexpected given that this species uses the site as a night roost. The lack of *M. velifer* samples was surprising, and we assume that this is because this species may deposit the bulk of its guano deeper in the cave, likely in sections where it roosts (Buecher & Sidner, 1999). At the bridge roost on Fort Huachuca, species representation was not at odds with known bat use (R. Sidner, unpublished data), but we did not detect a few species known to use the site at lower frequencies (e.g., *Choeronycteris mexicana* Tschudi, *Tadarida brasiliensis* I. Geoffroy). Greater numbers of samples would likely be needed to detect very rare species or those that infrequently deposit guano at the points where samples were collected.

One approach that can be employed to increase the amount of data obtained through MDM would be to assay more samples through a process of combining individual guano samples or DNA extracted from individual samples into one or more aggregate samples at some stage of sample processing or sequencing. Walker et al. (2019) recently demonstrated that the likelihood of detection for even very rare samples or DNA types (i.e., that might only occur in a single pellet) can be efficiently and effectively detected in this way. One concern with this approach is that extrapolating the relative frequencies of different taxa based on the frequency of encountering samples from each taxon may become less precise with aggregate samples (Mata et al., 2019).

The sex ratio estimate derived from MDM for the *M. lucifugus* roost on Fort Drum (100% female; Table 3) agreed with expectations, as this structure is a maternity roost and samples were taken prior to parturition and the presence of male pups (Wimsatt, 1945). Both *E. fuscus* samples from Fort Drum were also classified as female. Similarly, the female‐biased sex ratio obtained for *L. yerbabuenae* at Fort Huachuca's day roost was close to expectations based on the presence of adult females and weaned pups of both sexes (Fleming & Nassar, 2002; Hayward & Cockrum, 1971). Sex ratios for *M. velifer* at the bridge roost, which is not expected to have sex‐biased use, were more equal. The poor results for sex identification of *A. pallidus* and *L. yerbabuenae* samples collected from the bridge roost was likely due to a batch‐level human or instrument error, as we were able to arrive at sex identifications for 90% of *M. velifer* samples from the same roost, and for 74% and 88% *A. pallidus* and *L. yerbabuenae* samples from the day roost, respectively.

**TABLE 3:** Sex identification for bat species for guano samples taken from one two sites on Fort Huachuca, AZ

Fort Huachuca	Sex	*Antrozous pallidus*	*Leptonycteris yerbabuenae*	*Myotis velifer* or *Myotis yumanensis*	*Myotis thysanodes*
Cave	M	19	7	—	—
	F	16	28	—	—
	U	15	5	—	—
Bridge	M	1	1	57	0
	F	0	0	32	0
	U	33	19	10	2

Abbreviations: F, female; M, male; U, no identification.

The incidence of samples from Fort Drum containing DNA from *Pd* was well within expectations, given past WNS infection levels and previously documented presence of *Pd* at the colony (Dobony et al., 2011; Dobony & Johnson, 2018). Samples were collected in May 2016, about the time when the infection intensity and surface coverage of *Pd* on bat tissues in the region begins to drop (mid‐spring through summer; Langwig et al., 2015). DNA‐based detection of *Pd* in guano has the potential to be an important component of WNS monitoring, given that guano may be collected noninvasively. Additionally, recent studies have demonstrated that *Pd* DNA may be detected in guano during warmer periods after WNS is no longer observable on bats or detectable via DNA swabs of wings (Ballmann et al., 2017; Urbina et al., 2020). However, the extent to which the presence of *Pd* DNA in guano samples reflects colony WNS infection rates remains to be understood, and should be investigated.

Insect DNAs detected in *M. lucifugus* and *E. fuscus* samples from Fort Drum were similar to the results of diet studies for both species in the northeastern US and eastern Canada (Belwood & Fenton, 1976; Clare et al., 2011). Often hunting along the margins of water bodies or over water, *M. lucifugus* preys on insects associated with these habitats, especially those species characterized by mass emergences of flying adults (Anthony & Kunz, 1977; Belwood & Fenton, 1976; Buchler, 1976). In our dataset, the most commonly encountered prey groups included insects known to inhabit riparian areas, including *Chironomus* and other Diptera; *Callibaetis*, *Maccaffertium*, and *Caenis* within the Ephemeroptera; and Family Hydropsychidae within the Trichoptera. In some past studies, spiders were commonly identified among *M. lucifugus* dietary items (e.g., Feldhamer et al., 2009; Kaupas & Barclay, 2018; Shively et al., 2018; Whitaker & Lawhead, 1992). Several of the spider taxa represented in our dataset, including *Eris* (Family Salticidae), *Clubiona* (Family Clubionidae), and the most commonly encountered arachnid taxon, *Hibana* (Family Anyphaenidae), do not build or dwell in webs, but would have likely either been gleaned from vegetation or captured as ballooning spiderlings (Blandenier & Fürst, 1998; Dean & Sterling, 1985; Feldhamer et al., 2009; Ratcliffe & Dawson, 2003).

The putative prey ASVs obtained from the guano samples of primarily arthropod‐hunting bats from Fort Huachuca also aligned well with known diets. Hunting *A. pallidus* are known to consume fairly large, even venomous invertebrates, often gleaning prey off of plants and other surfaces (Hermanson & O'Shea, 1983; Johnston & Fenton, 2001). *Antrozous pallidus* samples in our study were dominated by large ground crickets and katydids (Orthoptera), and also included centipedes (Scolopendromorpha), mantids (Mantodea), and walking sticks (Phasmatodea). The most common prey item ASVs in samples from *M. velifer*/*M*. *yumanensis*, which primarily capture small insect prey in flight (Fitch et al., 1981), were from Coleoptera, which is a frequent dietary item for both species (Brigham et al., 1992; Kunz, 1974). Moths are part of the diets of most insectivorous bats, including *M. thysanodes* (Black, 1974), and the two *M. thysanodes* guano samples in our study contained DNA from Noctuid moths.

The diet of *L. yerbabuenae* is largely comprised of nectar, pollen, and fruit (Cole & Wilson, 2006; Edwards et al., 2019; Peñalba et al., 2006). During the sampling period of our study, *L. yerbabuenae* diet would be expected to consist almost entirely of *Agave* spp. (Cockrum, 1991; Fleming et al., 1993). The most frequently encountered plant ASVs from the combined *trnH*‐*psbA* and *18S rRNA* datasets among *L. yerbabuenae* splat samples were from Family Asparagaceae, which includes the subfamily containing *Agave* (Agavoideae). An interesting aspect of the *L. yerbabuenae* samples was that only about 28% (17 out of 60) contained plant ASVs, including only 16% of splat samples (8 of 39), but 43% (9 of 21) of pellet samples. The splat samples may have simply contained less DNA—17% of splats failed to provide bat species identification, compared to 5% failure of pellet samples (Fort Huachuca only), and 62% of splats failed to provide sex ASVs, compared to no failures for pellet samples (Fort Huachuca only). Another factor that might be affecting diet estimation from splats is that *L. yerbabuenae* may be regularly feeding on sugar water from hummingbird feeders (Buecher & Sidner, 2013; Fleming et al., 2021; Hinman, 2003), which likely contains little to no plant DNA.

The second most common plant ASV encountered in *L. yerbabuenae* samples was Family Myrtaceae. This family has several thousand recognized species and a worldwide distribution, with Old World species that are known to be pollinated by bats (Fleming et al., 2009), but no record of bat pollination in the New World. The congeneric *Leptonycteris curasoae*, which ranges in parts of northeastern South America and islands of the southwestern Caribbean Sea, is reported to consume the fruits of some Myrtaceae (Fleming & Nassar, 2002). It is possible that the Myrtaceae ASVs derived from bats consuming nectar or fruit from nonnative plants growing in local gardens, urban landscaping, or in the wild (Edwards et al., 2019). For example, plants in the genera *Eucalyptus*, *Myrtus*, *Callistemon*, and *Psidium* are commonly used in landscaping in southern Arizona. It is also possible that the DNA barcode loci, *trnH‐psbA* and *18SRNA*, were not sufficiently differentiated within Order Myrtales for classification to the family level. However, past studies have demonstrated species‐level, or even infraspecies‐level monophyly of the *trnH‐psbA* locus, including within subsets of Myrtaceae (Costion et al., 2011; Kress et al., 2015). Another explanation might be that the unanticipated ASVs derive from pollen “by‐catch” deposited by pollinators that have visited other plants within the local plant–pollinator network (Edwards et al., 2019; Lance et al., 2017). Additionally, wind‐dispersed pollen may simply be blown onto guano samples and be co‐processed with the collected sample.

A similar point of interest was the detection of numerous plant ASVs in the *18S rRNA* dataset obtained from bat species not known to be nectarivorous, herbivorous, or frugivorous. Though primarily a predator of arthropods and even small vertebrates, *A. pallidus* has also been observed to directly and/or incidentally feed on the nectar and fruit of columnar cacti and the nectar of *Agave* (Aliperti et al., 2017; Frick et al., 2009; Howell, 1980; Jaquish & Ammerman, 2021). This foraging strategy could account, at least in part, for the ASVs from Family Asparagaceae (containing *Agave*) and Order Caryophyllales (containing cacti) found in 25 and 11 *A. pallidus* samples, respectively. However, nearly equivalent numbers of samples contained ASVs from Asteraceae (*N* = 24), Poaceae (*N* = 18), and Fabaceae (*N* = 12). Additionally, several Caryophyllales ASVs found in *A. pallidus* samples were classified to non‐cactus groups, including Family Chenopodiaceae (amaranths) and Family Nyctaginaceae (four o'clocks). It seems just as likely then, that to a fairly large degree the plant DNA detected in the guano of *A. pallidus* originated either from pollen on the external surfaces of insect prey or from plant material within the guts of insect prey (Guenay et al., 2021; Sheppard et al., 2005). Likewise, ASVs from Fabaceae (*N* = 15 samples), Ericaceae (*N* = 12 samples), and other plants in *M. velifer*/*M. yumanensis* samples likely originated from indirect consumption of plant pollen and other tissues. Some ASVs, from families with wind‐dispersed pollen, such as Fagaceae, Pinaceae, and Poaceae, may have originated with wind‐blown pollen deposited on samples (though both collection sites were somewhat sheltered), insects, and plants.

The potential for DNA by‐catch data does not apply only to pollen or plant materials in insect gut, but also to the analysis of insect prey. Many of the prey items detected in samples from *A. pallidus* and *M. velifer*/*M. yumanensis* are species that spend considerable time on the ground (e.g., Corydiidae, Rhaphidophoridae, Tenebrionidae, *Onthophagus* [Scarabaeidae]), and/or that are predatory on other arthropods (e.g., Scolopendridae, Araneidae, and Mantidae), and it is conceivable that DNA from some arthropod ASVs originated from those insects crawling over samples already deposited on our collection tarps, or from material in the guts of predatory species. This and the other DNA by‐catch scenarios provide a caution that dietary data obtained from noninvasive, indirect tools like MDM should generally be interpreted very carefully, with full consideration of natural history information from prior studies. Additionally, for most metabarcoding applications, the performance of different enrichment approaches across different taxonomic groups (e.g., primer biases) and a priori criteria for how different factors will influence data interpretation (such as ASV read counts within samples and incidences of ASVs across samples), are key concerns (Deagle et al., 2019; Pompanon et al., 2012; Swift et al., 2018).

Finally, we detected ASVs from taxa known to be bat parasites (Duszynski et al., 1999; Jiménez et al., 2017; Peralta, 2012; Wheat, 1975) in the samples from both Fort Drum and Fort Huachuca, and with both the *18S rRNA* and *16S rRNA* barcode loci. These parasite ASVs included unicellular protists (e.g., Family Eimeriidae [Apicomplexa]), roundworms (e.g., Family Thelaziidae [Nematoda]), tapeworms (Family Hymenolepidae [Platyhelminthes]), flatworms (e.g., Family Lecithodendriidae [Platyhelminthes]), and fleas (e.g., Order Siphonoptera [Arthropoda]). The most commonly encountered parasite ASVs in the *M. lucifugus*, *A. pallidus*, *M. velifer*/*M. yumanensis*, and *M. thysanodes* samples were alveolates of the Order Eucoccidiorida, particularly Family Eimeriidae. ASVs corresponding to these parasites were also common in *L. yerbabuenae* samples.

Several features of multiplexed high‐throughput sequencing approaches like MDM warrant additional study or consideration. For one, applying multiple DNA barcodes to each sample in a metabarcoding survey has been shown to enhance the breadth of taxa detected (Aizpurua et al., 2018; Alberdi et al., 2018; da Silva et al., 2019; de Barba et al., 2014). In our case, we observed this effect with the *18S rRNA* barcode assay that, despite being included primarily to provide ASVs for endoparasites, also produced ASVs from insect prey and plant material. Likewise, we detected additional ASVs for parasites in the *16S* assay dataset, which was primarily intended to produce ASVs from insect prey. However, the number of loci to be multiplexed is a decision with trade‐offs. For example, the total number of reads that can be produced with each sequencing run is limited, based on the capability of the instrument. The more loci that are included in a sequencing run, all things being even remotely equal, the fewer reads that will be produced per locus. In addition to increasing number of loci, increasing the overall number of samples and increasing the number of replicate sequencing runs per sample can both enhance the diversity of taxa detected and/or the power to extrapolate relative frequencies of different targets (da Silva et al., 2019; Ficetola et al., 2015; Mata et al., 2019). Pilot studies are likely the best approach for determining the optimal number of loci to incorporate into MDM and, assuming some limits in available time, funds and/or supplies for sequencing and sequence analysis, for investigating important trade‐offs (discussed above) associated with that decision. When study goals are clearly defined, pilot studies utilizing even a few samples from the study system can explore the comparative numbers of unique ASVs detected and the depth of classification possible with different barcode loci and primer sets. These data would then enable researchers to effectively tailor the number and particular suite of loci to be used to achieve study goals.

## CONFLICT OF INTEREST

The authors declare that they have no conflict of interest.

## Supporting information


Figure S1

Table S1

Table S2

Table S3

Table S4

## Data Availability

All DNA sequences associated with amplicon sequencing variants detected as part of and pertinent to the findings of this study are openly available on Dryad at https://doi.org/10.5061/dryad.n8pk0p2xz, reference number n8pk0p2xz. Requests for additional data sharing may be directed to the corresponding author.
