# Supplementary figures and images for: Multifaceted DNA metabarcoding of guano to uncover multiple classes of ecological data in two different bat communities
Source: Evol Appl. 2022 Jun 29;15(7):1189–200. doi: 10.1111/eva.13425 (PMC9309442; doi:10.1111/eva.13425)

**SUPPLEMENTARY MATERIAL**

**Figure S1. MEGAN SETTINGS**


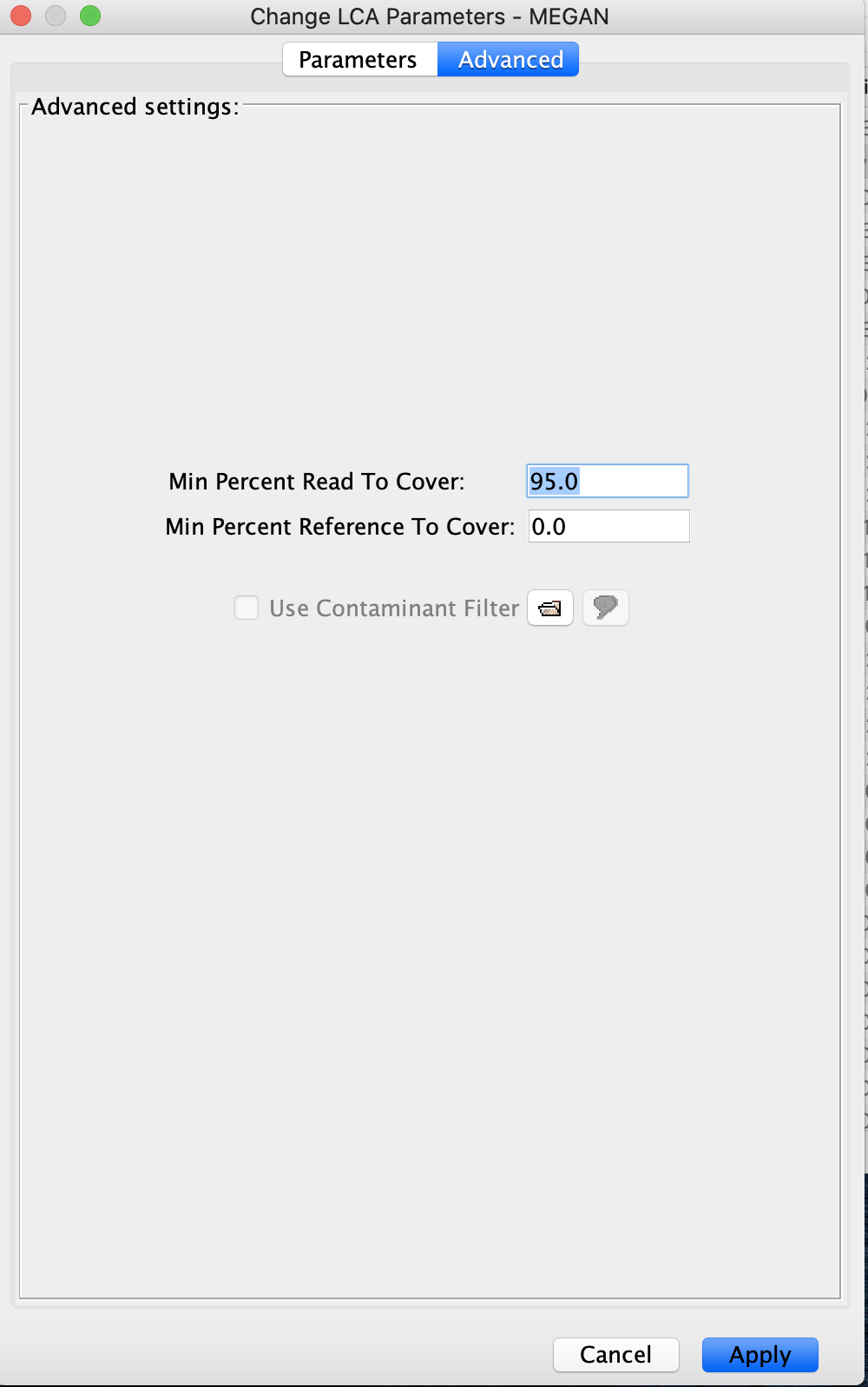

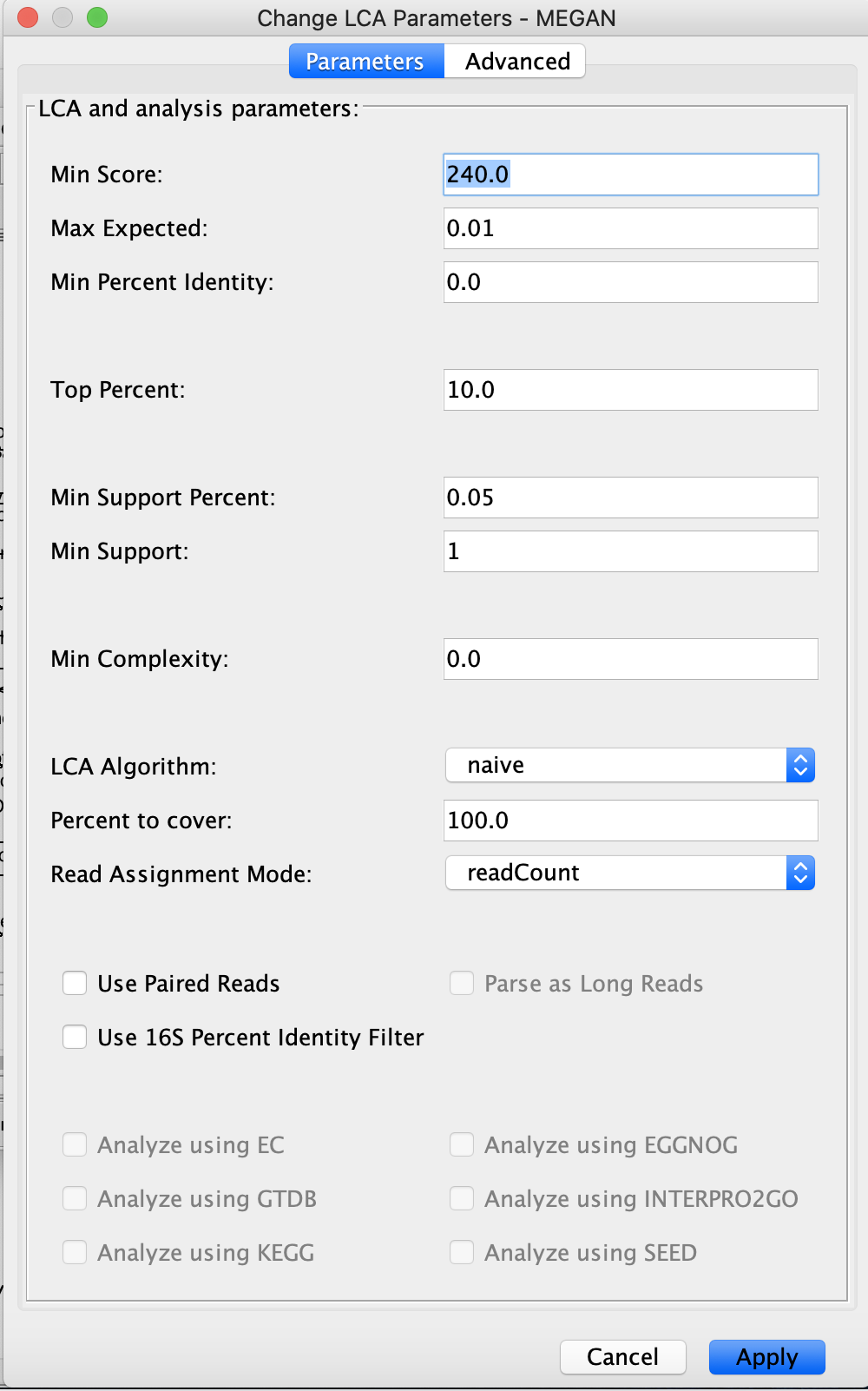

Supplement: Supplementary file 1 — Figure S1 [file EVA-15-1189-s004.docx]
