# Supplementary material for: Multifaceted DNA metabarcoding of guano to uncover multiple classes of ecological data in two different bat communities
Source: Evol Appl. 2022 Jun 29;15(7):1189–200. doi: 10.1111/eva.13425 (PMC9309442; doi:10.1111/eva.13425)
Supplement: Supplementary file 2 — Table S1 [file EVA-15-1189-s001.docx]

**S.1–S.4 DATA TABLES**

**Table S1.** *Multifaceted DNA Metabarcoding Next Generation Sequencing Metadata*. Key metrics from the seven 600-cycle, paired-end Illumina MiSeq next generation sequencing (NGS) runs employed for the reported multifaceted DNA metabarcoding (MDM) study, including the total numbers of guano samples included in each run (N_S_), the total raw, unmerged, and demultiplexed paired-end reads for each run (RUDm-Rds), the total number of reads in each run that could not be placed into a sample bin (no sample bin reads; ^1^NSB-Rds), and the total numbers of paired, aligned, and merged reads for each run (Mrgd-Rds). Each run also included two no-template control “samples” (NTCs). Also provided are key metrics for each locus (Locus/Primer Set) incorporated into each NGS run, including the total number of paired, aligned, and merged reads binned to that locus (N_Rds_), the mean (MN_Rds_) and standard deviation (SD) for reads assigned to each sample for that locus, the total number of reads (NTC_Rds_) found within the NTCs, the minimum and maximum number of reads (^2^NTC_Min_, NTC_Max_) found in the NTCs, the total number of “of interest” amplified sequence variants (ASVs) detected (^3^N_ASV_), and the mean (MN_ASV_) and standard deviation of such ASVs detected per sample. M1–M6 refer to different sets of six microsatellite loci, targeting *Myotis lucifugus* (Fort Drum samples) and *Leptonycteris yerbabuenae* (Fort Huachuca samples), respectively, that were initially included in the MDM-NGS effort, but were dropped from study due to poor data quality.

| **Ft. Drum** | **N_S_** | **RUDm-Rds** | **NSB-Rds** | **Mrgd-Rds** | **Locus/**  **Primer Set** | **N_Rds_** | **MN_Rds_**  **(SD)** | **NTC_Rds_** | **NTC_Min_**  **NTC_Max_** | **N_ASV_** | **MN_ASV_**  **(SD)** |
| --- | --- | --- | --- | --- | --- | --- | --- | --- | --- | --- | --- |
| Run 1 | 94 | 34.5M | 3.9M | 26.1M | M1–M6 | 17.5M^4^ | NA | NA | NA | NA | NA |
|  |  |  |  |  | *16S rRNA* | 6.00M | 63.80K (53.23K) | 64.98K | 28.25K  36.73K | 11.9K | 126.3  (64.9) |
|  |  |  |  |  | *Pd* ITS^5^ | 0.28M | 2.95K  (2.69K) | 0.43K | 0.17K 0.26K | 1.0K | 10.3 (5.1) |
|  |  |  |  |  | Y-chrom^6^ | 0.01M | 0.13K  (0.19K) | 0.28K | 0.01K  0.27K | 0.4K | 3.9  (4.3) |
|  |  |  |  |  | X-chrom^7^ | 1.02M | 10.88K  (8.96K) | 0.38K | 0.02K  0.35K | 1.3K | 13.7  10.6 |
|  |  |  |  |  | *18s rRNA* | 1.25M | 13.27K  (7.16K) | 0.27K | 0.03K  0.24K | 28.1K | 298.5 (151.2) |
|  |  |  |  |  |  |  |  |  |  |  |  |
| Run 2 | 94 | 34.0M | 3.3M | 23.7M | M1–M6 | 25.0M | NA | NA | NA | NA | NA |
|  |  |  |  |  | *16S rRNA* | 6.89M | 73.33K (30.84K) | 134.37K | 67.08K  67.30K | 12.8K | 137.5 (77.1) |
|  |  |  |  |  | *Pd* ITS | 0.22M | 2.37K  (1.78K) | 0.73K | 0.32K  0.41K | 0.7K | 7.4 (4.8) |
|  |  |  |  |  | Y-chrom | 0.05M | 0.55K  (1.28K) | 37.25K | 17.81K  19.45K | 0.4K | 4.2  (5.4) |
|  |  |  |  |  | X-chrom | 0.90M | 9.57K  (11.65K) | 35.87K | 17.15K  18.72K | 0.7K | 7.6  (8.4) |
|  |  |  |  |  | *18s rRNA* | 0.92M | 9.78K  (8.98K) | 2.90K | 1.22K  1.67K | 24.8K | 266.9 (158.2) |
|  |  |  |  |  |  |  |  |  |  |  |  |
| Run 3 | 94 | 24.2M | 3.8M | 21.3M | M1–M6 | 18.6M | NA | NA | NA | NA | NA |
|  |  |  |  |  | *16S rRNA* | 3.88M | 41.30K (29.35K) | 127.23K | 50.82K  76.42K | 3.3K | 37.1 (25.3) |
|  |  |  |  |  | *Pd* ITS | 0.31M | 3.31K  (2.83K) | 0.04K | 0.02K  0.02K | 1.1K | 11.4 (8.2) |
|  |  |  |  |  | Y-chrom | 0.22M | 0.23K  (0.26K) | 0.42K | 0.01K  0.41K | 0.6K | 6.0  (6.6) |
|  |  |  |  |  | X-chrom | 0.91M | 9.66K  (9.50K) | 0.80K | 0.02K  0.78K | 1.6K | 17.0  (17.1) |
|  |  |  |  |  | *18s rRNA* | 0.28M | 2.96K  (3.84K) | 2.85K | 0.03K  2.82K | 9.2K | 98.3 (108.4) |
|  |  |  |  |  |  |  |  |  |  |  |  |
|  |  |  |  |  |  |  |  |  |  |  |  |
| Run 4 | 94 | 27.7M | 3.6M | 22.2M | M1–M6 | 22.1M | NA | NA | NA | NA | NA |
|  |  |  |  |  | *16S rRNA* | 3.19M | 34.33K (16.88K) | 95.78K | 43.08K  52.70K | 3.8K | 40.9 (26.4) |
|  |  |  |  |  | *Pd* ITS | 0.43M | 4.62K  (5.09K) | 1.55K | 0.65K  0.90K | 1.4K | 14.3 (8.2) |
|  |  |  |  |  | Y-chrom | 0.21M | 0.22K  (0.30K) | >0.01K | 0.002K  0.002K | 0.6K | 6.7  (6.4) |
|  |  |  |  |  | X-chrom | 1.28M | 13.90K  (9.87K) | 0.17K | 0.09K  0.09K | 1.7K | 18.1  (13.5) |
|  |  |  |  |  | *18s rRNA* | 0.46M | 4.94K  (4.13K) | 8.33K | 4.13K  4.21K | 15.0K | 159.7 (85.6) |
|  |  |  |  |  |  |  |  |  |  |  |  |
| **Ft. Huachuca** | **N_S_** | **RUDm-Rds** | **NSB-Rds** | **Mrgd-Rds** | **Locus/**  **Primer Set** | **N_Rds_** | **MN_Rds_**  **(SD)** | **NTC_Rds_** | **NTC_Min_**  **NTC_Max_** | **N_ASV_** | **MN_ASV_**  **(SD)** |
| Run 1 | 94 | 26.1M | 2.4M | 14.0M | M1–M6 | 9.37M | NA | NA | NA | NA | NA |
|  |  |  |  |  | *16S rRNA* | 2.17M | 23.04K  (16.54K) | 0.15K | 0.06K  0.09K | 1.41K | 14.7  (11.8) |
|  |  |  |  |  | *trnH*–*psbA* | 0.62M | 6.54K  (8.62K) | 0.19K | 0.06K  0.12K | 3.45K | 36.0  (40.8) |
|  |  |  |  |  | Y-chrom | 0.02M | 0.25K  (0.46K) | 1.10K | 0.08K  0.98K | 1.70K | 17.7  (22.5) |
|  |  |  |  |  | X-chrom | 0.07M | 0.78K  (1.93K) | 2.56K | 0.39K  2.18K | 8.34K | 86.8  (170.0) |
|  |  |  |  |  | Y-chrom^8^ | 0.41M | 4.39K  (4.52K) | 0.38K | 0.13K  0.25K | 9.24K | 96.3  (84.7) |
|  |  |  |  |  | X-chrom^9^ | 0.11M | 1.21K  (1.32K) | 0.21K | 0.05K  0.16K | 4.48K | 46.6  (46.3) |
|  |  |  |  |  | *18s rRNA* | 1.23M | 13.18K  (11.31K) | 1.22K | 0.59K  0.62K | 0.18K | 1.8  (4.1) |
|  |  |  |  |  | Y-chrom^10^ | 0.00M | 0.00K | 0.00K | 0.00K | AFE^13^ | AFE |
|  |  |  |  |  | X-chrom^11^ | 0.0004M | 0.004K  (0.02K) | 0.00K | 0.00K | AFE^13^ | AFE |
|  |  |  |  |  |  |  |  |  |  |  |  |
| Run 2 | 94 | 24.8M | 2.5M | 16.4M | M1–M6 | 7.27M | NA | NA | NA | NA | NA |
|  |  |  |  |  | *16S rRNA* | 5.98M | 63.58K  (41.89K) | 2.80K | 1.39K  1.41K | 3.53K | 36.8  (23.1) |
|  |  |  |  |  | *trnH*–*psbA* | 0.89M | 9.44K  (11.09K) | 1.10K | 0.53K  0.57K | 22.5K | 234.2  (275.2) |
|  |  |  |  |  | Y-chrom | 0.03M | 0.31K  (0.95K) | 6.38K | 0.06K  6.32K | 1.3K | 12.9  (32.5) |
|  |  |  |  |  | X-chrom | 0.03M | 0.30K  (0.77K) | 7.42K | 0.07K  7.35K | 0.6K | 6.6  (11.9) |
|  |  |  |  |  | Y-chrom | 0.02M | 0.17K  (0.51K) | 0.06K | 0.02K  0.04K | 0.23K | 2.4  (2.9) |
|  |  |  |  |  | X-chrom | 0.01M | 0.08K  (0.15K) | 0.21K | 0.07K  0.14K | 0.16K | 1.7  (3.4) |
|  |  |  |  |  | *18s rRNA* | 2.13M | 22.70K  (15.07K) | 13.81K | 5.20K  8.61K | 27.2K | 283.8  (284.0) |
|  |  |  |  |  | Y-chrom | 0.006M | 0.06K  (0.41K) | 0.006K | 0.003K  0.003K | 0.2K | 2.0  (10.6) |
|  |  |  |  |  | X-chrom | 0.03M | 0.27K  (1.03K) | 0.005K | 0.002K  0.003K | 1.7K | 18.1  (64.0) |
|  |  |  |  |  |  |  |  |  |  |  |  |
| Run 3 | 94^12^ | 25.0M | 1.9M | 13.8M | M1–M6 | 5.44M | NA | NA | NA | NA | NA |
|  |  |  |  |  | *16S rRNA* | 3.87M | 41.18K  (33.25K) | 11.43K | 3.91K  7.52K | 2.56K | 26.7  (21.3) |
|  |  |  |  |  | *trnH*–*psbA* | 1.02M | 10.79K  (11.88K) | 3.78K | 1.04K  2.74K | 18.9K | 196.9  (237.3) |
|  |  |  |  |  | Y-chrom | 0.05M | 0.56K  (1.21K) | 0.35K | 0.03K  0.33K | 3.46K | 36.0  (41.2) |
|  |  |  |  |  | X-chrom | 0.01M | 1.40K  (2.98K) | 0.80K | 0.01K  0.79K | 14.8K | 77.3  (206.0) |
|  |  |  |  |  | Y-chrom | 1.35M | 14.32K  (17.41K) | 3.48K | 0.40K  3.08K | 36.5K | 380.7  (347.3) |
|  |  |  |  |  | X-chrom | 0.26M | 2.80K  (4.22K) | 0.48K | 0.15K  0.33K | 16.0K | 166.2  (209.0) |
|  |  |  |  |  | *18s rRNA* | 1.08M | 11.53K  (10.08K) | 11.42K | 5.41K  6.01K | 12.8K | 133.4  (159.1) |
|  |  |  |  |  | Y-chrom | 0.05M | 0.50K  (0.55K) | 0.006K | 0.002K  0.004K | 5.7K | 59.3  (58.6) |
|  |  |  |  |  | X-chrom | 0.67M | 7.12K  (10.14K) | 0.10K | 0.04K  0.05K | 57.1K | 594.8  (688.0) |

^1^The total number of reads binned as “undetermined” as opposed to assigned to one of the possible 94 samples or two no-treatment controls, as a result of an amplicon lacking one or both anticipated multiplex identifier (or index) sequences.

^2^As only two NTCs were incorporated into each run, the minimum and maximum values are more useful metrics than the mean and standard deviation.

^3^ASVs corresponding to taxa that were not components of the study and/or that are common NGS contaminant DNA sources, such as bacteria, house mice, and humans, were not included in these read count statistics.

^4^For each run, N_Rds_ for the combined six microsatellite primer sets is calculated as the difference between Mrgd-Rds and the sum of N_Rds_ for all other primer sets.

^5^*Pd* = *Pseudogymnoascus destructans*; ITS = internal transcribed spacer region locus.

^6^The Y-chromosome marker is located in the *Zinc Finger-Y* (*ZFY*) region and, in this case, assayed using the XGYC primers.

^7^The X-chromosome marker is located in the *Zinc Finger-X* (*ZFX*) region and, in this case, assayed using the XGXC primers.

^8^The Y-chromosome marker is located in the *Zinc Finger-Y* (*ZFY*) region and, in this case, assayed using the KYZF primers.

^9^The X-chromosome marker is located in the *Zinc Finger-X* (*ZFX*) region and, in this case, assayed using the KXZF primers.

^10^The Y-chromosome marker is located in the *Zinc Finger-Y* (*ZFY*) region and, in this case, assayed using the XGYC.ly primers (XGYC primers that are sequence-optimized for *L. yerbabuenae*).

^11^The X-chromosome marker is located in the *Zinc Finger-X* (*ZFX*) region and, in this case, assayed using the XGXC.ly primers (XGXC primers that are sequence-optimized for *L. yerbabuenae*).

^12^In addition to 86 previously unassayed guano samples, this run included a set of eight randomly-selected samples that had also been included in the prior two MDM NGS runs.

^13^AFE = apparently failed execution. No data remained following processing of sequencing reads through pipeline, likely due to technician error or instrument failure during library enrichment and/or preparation for this set of samples.
