# Supplementary material for: Multifaceted DNA metabarcoding of guano to uncover multiple classes of ecological data in two different bat communities
Source: Evol Appl. 2022 Jun 29;15(7):1189–200. doi: 10.1111/eva.13425 (PMC9309442; doi:10.1111/eva.13425)
Supplement: Supplementary file 3 — Table S2 [file EVA-15-1189-s003.docx]

**Table S2.** *NTC Read Counts*. The highest number of reads observed for amplified sequence variants (ASVs) of interest in no-template control (NTC) sequence datasets produced as part of an MDM (multifaceted DNA metabarcoding) study of bat guano collected from roosts on Fort Drum, NY and Fort Huachuca, AZ. The highest number of reads for each ASV in the NTCs was used as a guide for setting the 2-3X read count cut-off for retaining guano sample ASVs. If highest read count data is not provided for a locus, no ASVs of interest were observed for that locus in the NTCs. Highest read counts for those ASVs that would not impact study results (e.g., DNA barcode sequences classified as *Mus musculus* or *Homo sapiens*) are not reported. ASVs are reported by corresponding DNA locus, and by Illumina MiSeq run. Each sequencing run included one NTC sample (e.g., FD1 or FH1), including separate sequence datasets for each of the paired-end sequencing orientations (e.g. FD1.1 or FD1.2). The *16S rRNA* DNA barcode locus was utilized for determination of bat species, as well as for characterization of bat prey and parasite ASVs. The 18S *rRNA* DNA barcode locus was utilized for characterization of parasite, prey, and plant ASVs.

|  | **Sequencing Runs and Reads** | | | | | | | |
| --- | --- | --- | --- | --- | --- | --- | --- | --- |
| **Fort Drum Bat Guano ASVs** | **FD1.1** | **FD1.2** | **FD2.1** | **FD2.2** | **FD3.1** | **FD3.2** | **FD4.1** | **FD4.2** |
| ***16S rRNA*** |  |  |  |  |  |  |  |  |
| *Myotis lucifugus* | 190 | 122 | 59 | 42 | 39 | 31 | 43 | 42 |
| *Eptesicus fuscus* | 0 | 0 | 0 | 0 | 0 | 0 | 3 | 3 |
| *Caloca ascita* | 10 | 8 | 0 | 2 | 0 | 3 | 6 | 0 |
| *Symplecta hybrida* | 3 | 2 | 0 | 0 | 0 | 0 | 0 | 0 |
| *Maccaffertium smithae* | 4 | 0 | 0 | 0 | 0 | 0 | 0 | 0 |
| *Maccaffertium vicarium* | 0 | 4 | 0 | 0 | 0 | 0 | 0 | 0 |
| *Stenonema femoratum* | 5 | 4 | 0 | 0 | 0 | 0 | 0 | 0 |
| *Choroterpes* sp. GG-2004 | 0 | 0 | 3 | 6 | 0 | 0 | 0 | 0 |
| *Leptohyphes zalope* | 0 | 0 | 0 | 0 | 3 | 2 | 0 | 0 |
| *Stenacron palladium* | 0 | 0 | 6 | 3 | 0 | 0 | 2 | 2 |
| *Serica* sp. | 2 | 2 | 0 | 0 | 0 | 0 | 0 | 0 |
|  |  |  |  |  |  |  |  |  |
| ***18S rRNA*** |  |  |  |  |  |  |  |  |
| Eimeriidae | 0 | 4 | 6 | 0 | 0 | 0 | 0 | 0 |
|  |  |  |  |  |  |  |  |  |
| **Sex Chromosomes** |  |  |  |  |  |  |  |  |
| XGXC | 10 | 0 | 0 | 0 | 7 | 4 | 0 | 0 |
| XGYC | 0 | 0 | 0 | 0 | 0 | 0 | 0 | 0 |
|  |  |  |  |  |  |  |  |  |
|  |  |  |  |  |  |  |  |  |
| **Fort Huachuca Bat Guano ASVs** | **FH1.1** | **FH1.2** | **FH2.1** | **FH2.2** | **FH3.1** | **FH3.2** |  |  |
| ***16S rRNA*** |  |  |  |  |  |  |  |  |
| *Antrozous pallidus* | 0 | 0 | 17 | 7 | 2 | 3 |  |  |
| *Leptonycteris yerbabuenae* | 0 | 0 | 17 | 20 | 52 | 34 |  |  |
| *Myotis* sp. | 2 | 0 | 12 | 14 | 0 | 5 |  |  |
|  |  |  |  |  |  |  |  |  |
| ***18S rRNA*** |  |  |  |  |  |  |  |  |
| *Fictor* sp. | 0 | 0 | 2 | 0 | 0 | 0 |  |  |
