# Supplementary material for: Multifaceted DNA metabarcoding of guano to uncover multiple classes of ecological data in two different bat communities
Source: Evol Appl. 2022 Jun 29;15(7):1189–200. doi: 10.1111/eva.13425 (PMC9309442; doi:10.1111/eva.13425)
Supplement: Supplementary file 4 — Table S3 [file EVA-15-1189-s005.docx]

**Table S3.1.a.** *Eptesicus fuscus*. Numbers of *Eptesicus fuscus* guano samples from Fort Drum, NY containing *16S* *rRNA* and *18S* *rRNA* amplified sequence variants (ASVs) that were classified to likely prey items. N_C_ = number of guano samples containing ASVs classified to the corresponding class, N_O_ = number of guano samples containing ASVs classified to the corresponding order, N_F_ = number of guano samples containing ASVs classified to the corresponding family, and N_G_ = number of guano samples containing ASVs classified to the corresponding genus.

| **Class** | **N_C_** | **Order** | **N_O_** | **Family** | **N_F_** | **Genus** | **N_G_** | **Species** | **N_S_** |
| --- | --- | --- | --- | --- | --- | --- | --- | --- | --- |
| Insecta | 2 | Coleoptera | 2 | Curculionidae^16^ | 1 | *Hylobitelus*^16^ | 1 |  |  |
|  |  |  |  |  |  | *Hylobius*^16^ | 1 |  |  |
|  |  |  |  | Elateridae^16^ | 1 | *Melanotus*^16^ | 1 | *Melanotus similis*^16^ | 1 |
|  |  |  |  | Scarabaeidae^16^ | 1 |  |  |  |  |

^16^ Designates taxa found only in *16S* *rRNA* sequence dataset.

**Table S3.1.b.** *Myotis lucifugus*. Numbers of *M. lucifugus* guano samples from Fort Drum, NY containing *16S* *rRNA* and *18S* *rRNA* amplified sequence variants (ASVs) that were classified to likely prey items. N_C_ = number of guano samples containing ASVs classified to the corresponding class, N_O_ = number of guano samples containing ASVs classified to the corresponding order, N_F_ = number of guano samples containing ASVs classified to the corresponding family, N_G_ = number of guano samples containing ASVs classified to the corresponding genus, and N_S_ = number of guano samples containing ASVs classified to the corresponding species.

| **Class** | **N_C_** | **Order** | **N_O_** | **Family** | **N_F_** | **Genus** | **N_G_** | **Species** | **N_S_** |
| --- | --- | --- | --- | --- | --- | --- | --- | --- | --- |
| Arachnida | 95 | Aranae | 64 | Agelenidae^16^ | 1 | *Agelenopsis*^16^ | 1 |  |  |
|  |  |  |  | Anyphaenidae^16^ | 31 | *Hibana*^16^ | 31 |  |  |
|  |  |  |  | Araneidae^16^ | 6 | *Larinioides*^16^ | 4 | *Larinioides cornutus*^16^ | 3 |
|  |  |  |  |  |  |  |  | *Larinioides sclopetarius*^16^ | 1 |
|  |  |  |  | Clubionidae^16^ | 9 | *Clubiona*^16^ | 4 |  |  |
|  |  |  |  | Linyphiidae | 9 | *Pityohyphantes*^16^ | 6 | *Pityohyphantes costatus*^16^ | 4 |
|  |  |  |  | Lycosidae^16^ | 14 |  |  |  |  |
|  |  |  |  | Mimetidae^16^ | 5 | *Mimetus*^16^ | 5 |  |  |
|  |  |  |  | Philodromidae^16^ | 3 |  |  |  |  |
|  |  |  |  | Salticidae^16^ | 1 | *Eris*^16^ | 1 | *Eris militaris*^16^ | 1 |
|  |  |  |  | Tetragnathidae^16^ | 6 | *Tetragnatha*^16^ | 6 | *Tetragnatha laboriosa*^16^ | 1 |
|  |  |  |  | Theridiidae^16^ | 5 | *Enoplognatha*^16^ | 1 |  |  |
|  |  |  |  |  |  | *Keijia*^16^ | 1 | *Keijia alabamensis*^16^ | 1 |
|  |  |  |  |  |  | *Theridion*^16^ | 3 | *Theridion murarium*^16^ | 3 |
|  |  |  |  | Thomisidae^16^ | 1 | *Xysticus*^16^ | 1 |  |  |
|  |  |  |  | Zodariidae^16^ | 2 |  |  |  |  |
|  |  | Opiliones | 1 | Phalangiidae^16^ | 1 |  |  |  |  |
| Insecta | 298 | Coleoptera | 29 | Cantharidae^16^ | 1 |  |  |  |  |
|  |  |  |  | Chrysomelidae^16^ | 1 |  |  |  |  |
|  |  |  |  | Cleridae^16^ | 1 |  |  |  |  |
|  |  |  |  | Curculionidae^16^ | 3 | *Phyllobius*^16^ | 2 |  |  |
|  |  |  |  | Dermestidae^16^ | 1 | *Anthrenus*^16^ | 1 |  |  |
|  |  |  |  | Dytiscidae^16^ | 1 |  |  |  |  |
|  |  |  |  | Elateridae^16^ | 2 | *Aeolus*^16^ | 1 | *Aeolus mellillus*^16^ | 1 |
|  |  |  |  |  |  | *Melanotus*^16^ | 1 | *Melanotus similis*^16^ | 1 |
|  |  |  |  | Hydraenidae^16^ | 2 |  |  |  |  |
|  |  |  |  | Hydrophilidae^16^ | 3 |  |  |  |  |
|  |  |  |  | Oedemeridae^16^ | 1 |  |  |  |  |
|  |  |  |  | Phalacridae^16^ | 12 |  |  |  |  |
|  |  |  |  | Scarabaeidae^16^ | 5 | *Phyllophaga*^16^ | 1 |  |  |
|  |  |  |  |  |  | *Serica*^16^ | 1 |  |  |
|  |  |  |  | Silvanidae^16^ | 1 |  |  |  |  |
|  |  |  |  | Staphylinidae^16^ | 2 |  |  |  |  |
|  |  | Diptera | 183 | Acroceridae^16^ | 1 | *Ogcodes*^16^ | 1 |  |  |
|  |  |  |  | Axymyiidae^16^ | 2 |  |  |  |  |
|  |  |  |  | Bibionidae^16^ | 2 | *Bibio*^16^ | 1 |  |  |
|  |  |  |  |  |  | *Dilophus*^16^ | 1 |  |  |
|  |  |  |  | Calliphoridae^16^ | 3 |  |  |  |  |
|  |  |  |  | Cecidomyiidae^16^ | 1 |  |  |  |  |
|  |  |  |  | Ceratopogonidae^16^ | 6 | *Bezzia*^16^ | 2 |  |  |
|  |  |  |  | Chironomidae^16^ | 148 | *Chironomus*^16^ | 137 |  |  |
|  |  |  |  |  |  | *Diamesa*^16^ | 1 |  |  |
|  |  |  |  |  |  | *Micropsectra*^16^ | 5 | *Micropsectra insignilobus*^16^ | 5 |
|  |  |  |  |  |  | *Paratanytarsus*^16^ | 2 | *Paratanytarsus tenuis*^16^ | 2 |
|  |  |  |  |  |  | *Parochlus*^16^ | 8 |  |  |
|  |  |  |  |  |  | *Tanytarsus*^16^ | 5 | *Tanytarsus mendax*^16^ | 4 |
|  |  |  |  | Culicidae^16^ | 5 |  |  |  |  |
|  |  |  |  | Diopsidae^16^ | 1 |  |  |  |  |
|  |  |  |  | Dixidae^16^ | 2 | *Dixella*^16^ | 2 |  |  |
|  |  |  |  | Dolichopodidae^16^ | 4 |  |  |  |  |
|  |  |  |  | Drosophilidae^16^ | 10 |  |  |  |  |
|  |  |  |  | Empididae^16^ | 2 | *Hilara*^16^ | 2 |  |  |
|  |  |  |  | Ephydridae^16^ | 1 | *Hydrellia*^16^ | 1 |  |  |
|  |  |  |  | Limoniidae^16^ | 32 | *Rhipidia*^16^ | 1 |  |  |
|  |  |  |  |  |  | *Symplecta*^16^ | 28 | *Symplecta hybrida*^16^ | 28 |
|  |  |  |  | Mycetophilidae^16^ | 3 | *Phronia*^16^ | 1 | *Phronia strenua*^16^ | 1 |
|  |  |  |  | Pediciidae^16^ | 6 | *Pedicia*^16^ | 1 |  |  |
|  |  |  |  | Phoridae^16^ | 1 | *Borophaga*^16^ | 1 | *Borophaga verticalis*^16^ | 1 |
|  |  |  |  | Psychodidae^16^ | 20 | *Psychomora*^16^ | 20 |  |  |
|  |  |  |  | Sciaridae^16^ | 6 | *Bradysia*^16^ | 5 |  |  |
|  |  |  |  | Simuliidae^16^ | 2 | *Simulium*^16^ | 2 |  |  |
|  |  |  |  | Syrphidae^16^ | 1 |  |  |  |  |
|  |  |  |  | Tabanidae^16^ | 1 |  |  |  |  |
|  |  |  |  | Tachinidae^16^ | 3 | *Blondelia*^16^ | 3 | *Blondelia nigripes*^16^ | 1 |
|  |  |  |  | Tephritidae^16^ | 1 |  |  |  |  |
|  |  |  |  | Tipulidae^16^ | 8 | *Tipula*^16^ | 8 |  |  |
|  |  |  |  | Trichoceridae^16^ | 23 | *Trichocera*^16^ | 23 | *Trichocera brevicornis*^16^ | 23 |
|  |  |  |  | Ulidiidae^16^ | 1 |  |  |  |  |
|  |  | Ephemeroptera | 150 | Baetidae^16^ | 69 | *Callibaetis*^16^ | 66 | *Callibaetis ferrugineus*^16^ | 66 |
|  |  |  |  |  |  | *Cloeon*^16^ | 17 | *Cloeon dipterum*^16^ | 17 |
|  |  |  |  | Caenidae^16^ | 45 | *Caenis*^16^ | 45 |  |  |
|  |  |  |  | Ephemerellidae^16^ | 5 |  |  |  |  |
|  |  |  |  | Ephemeridae^16^ | 2 | *Ephemera*^16^ | 2 | *Ephemera simulans*^16^ | 2 |
|  |  |  |  | Heptageniidae^16^ | 65 | *Epeorus*^16^ | 2 | *Epeorus vitreus*^16^ | 2 |
|  |  |  |  |  |  | *Maccaffertium*^16^ | 54 | *Maccaffertium ithaca*^16^ | 29 |
|  |  |  |  |  |  |  |  | *Maccaffertium smithae*^16^ | 23 |
|  |  |  |  |  |  |  |  | *Maccaffertium vicarium*^16^ | 11 |
|  |  |  |  |  |  | *Rhithrogena*^16^ | 6 |  |  |
|  |  |  |  |  |  | *Stenonema*^16^ | 9 | *Stenonema femoratum*^16^ | 9 |
|  |  |  |  | Leptohyphidae^16^ | 1 |  |  |  |  |
|  |  |  |  | Leptophlebiidae^16^ | 6 | *Choroterpes*^16^ | 6 |  |  |
|  |  | Hemiptera^16^ | 19 | Achilidae^16^ | 1 |  |  |  |  |
|  |  |  |  | Adelgidae^16^ | 2 | *Adelges*^16^ | 2 |  |  |
|  |  |  |  | Aphididae^16^ | 1 | *Drepanaphis*^16^ | 1 |  |  |
|  |  |  |  | Aphrophoridae^16^ | 1 |  |  |  |  |
|  |  |  |  | Berytidae^16^ | 1 |  |  |  |  |
|  |  |  |  | Cicadellidae^16^ | 3 | *Homalodisca*^16^ | 1 |  |  |
|  |  |  |  | Corixidae^16^ | 5 | *Glaenocorisa*^16^ | 5 | *Glaenocorisa propinqua*^16^ | 1 |
|  |  |  |  | Issidae^16^ | 1 |  |  |  |  |
|  |  |  |  | Miridae^16^ | 1 |  |  |  |  |
|  |  |  |  | Pemphigidae^16^ | 2 | *Pemphigus*^16^ | 1 |  |  |
|  |  |  |  | Psyllidae^16^ | 2 |  |  |  |  |
|  |  | Hymenoptera |  | Braconidae^16^ | 7 |  |  |  |  |
|  |  |  |  | Cephidae | 1 | *Janus*^16^ | 1 |  |  |
|  |  |  |  | Formicidae | 2 |  |  |  |  |
|  |  |  |  | Ichneumonidae^16^ | 1 |  |  |  |  |
|  |  |  |  | Megastigmidae^16^ | 1 |  |  |  |  |
|  |  |  |  | Vespidae^16^ | 1 |  |  |  |  |
|  |  | Lepidoptera | 26 | Crambidae^16^ | 3 |  |  |  |  |
|  |  |  |  | Gelechiidae^16^ | 2 |  |  |  |  |
|  |  |  |  | Geometridae^16^ | 3 | *Ectropis*^16^ | 1 |  |  |
|  |  |  |  |  |  | *Operophtera*^16^ | 1 |  |  |
|  |  |  |  | Gracillariidae^16^ | 3 | *Phyllonorycter*^16^ | 2 |  |  |
|  |  |  |  | Limacodidae^16^ | 3 |  |  |  |  |
|  |  |  |  | Noctuidae^16^ | 1 |  |  |  |  |
|  |  |  |  | Nymphalidae^16^ | 1 |  |  |  |  |
|  |  |  |  | Papilionidae^16^ | 1 |  |  |  |  |
|  |  |  |  | Plutellidae^16^ | 2 | *Plutella*^16^ | 1 | *Plutella xylostella*^16^ | 1 |
|  |  |  |  | Tortricidae^16^ | 6 | *Acleris*^16^ | 3 |  |  |
|  |  | Mecoptera^16^ | 1 | Panorpidae^16^ | 1 |  |  |  |  |
|  |  | Megaloptera^16^ | 4 | Corydalidae^16^ | 1 |  |  |  |  |
|  |  |  |  | Sialidae^16^ | 3 | *Sialis*^16^ | 3 |  |  |
|  |  | Neuroptera | 16 | Chrysopidae^16^ | 1 |  |  |  |  |
|  |  |  |  | Coniopterygidae | 2 | *Coniopteryx*^16^ | 1 |  |  |
|  |  |  |  |  |  | *Conwentzia*^16^ | 1 | *Conwentzia pineticola*^16^ | 1 |
|  |  |  |  | Hemerobiidae | 14 | *Hemerobius*^16^ | 2 | *Hemerobius stigma*^16^ | 2 |
|  |  | Odonata^16^ | 1 | Libellulidae^16^ | 1 | *Tetrathemis*^16^ | 1 | *Tetrathemis polleni*^16^ | 1 |
|  |  | Orthoptera | 4 | Gryllidae^16^ | 1 | *Eunemobius*^16^ | 1 | *Eunemobius carolinus*^16^ | 1 |
|  |  |  |  | Tettigoniidae^16^ | 3 |  |  |  |  |
|  |  | Plecoptera | 16 | Leuctridae^16^ | 10 | *Leuctra*^16^ | 10 |  |  |
|  |  |  |  | Perlodidae^16^ | 5 | *Helopicus*^16^ | 2 |  |  |
|  |  |  |  |  |  | *Isoperla*^16^ | 1 |  |  |
|  |  | Psocoptera^16^ | 7 | Ectopsocidae^16^ | 1 | *Ectopsocopsis*^16^ | 1 | *Ectopsocopsis cryptomeriae*^16^ | 1 |
|  |  |  |  | Peripsocidae^16^ | 1 | *Peripsocus*^16^ | 1 | *Peripsocus madidus*^16^ | 1 |
|  |  |  |  | Psocidae^16^ | 6 | *Blaste*^16^ | 2 | *Blaste quieta*^16^ | 2 |
|  |  |  |  |  |  | *Metylophorus*^16^ | 5 | *Metylophorus novaescotiae*^16^ | 5 |
|  |  |  |  |  |  | *Trichadenotecnum*^16^ | 1 | *Trichadenotecnum slossonae*^16^ | 1 |
|  |  | Thysanoptera^16^ | 4 | Thripidae^16^ | 4 | *Anaphothrips*^16^ | 2 | *Anaphothrips obscurus*^16^ | 2 |
|  |  | Trichoptera | 159 | Hydropsychidae^16^ | 63 | *Cheumatopsyche*^16^ | 9 |  |  |
|  |  |  |  |  |  | *Hydropsyche*^16^ | 2 |  |  |
|  |  |  |  | Limnephilidae^16^ | 3 | *Limnephilus*^16^ | 2 |  |  |
|  |  |  |  | Phryganeidae^16^ | 4 | *Agrypnia*^16^ | 3 |  |  |
|  |  |  |  | Rhyacophilidae^16^ | 1 | *Rhyacophila*^16^ | 1 |  |  |

^16^ Designates taxa found only in *16S* *rRNA* sequence dataset.

^18^ Designates taxa found only in *18S* *rRNA* sequence dataset.

**Table S3.2.a.** *Antrozous pallidus*. Numbers of *A. pallidus* guano samples from Fort Huachuca, AZ, containing *16S* *rRNA* and *18S* *rRNA* amplified sequence variants (ASVs) that were classified to likely prey items. N_C_ = number of guano samples containing ASVs classified to the corresponding class, N_O_ = number of guano samples containing ASVs classified to the corresponding order, and N_F_ = number of guano samples containing ASVs classified to the corresponding family.

| **Class** | **N_C_** | **Order** | **N_O_** | **Family** | **N_F_** |
| --- | --- | --- | --- | --- | --- |
| Chilopoda | 5 | Scolopendromorpha | 5 | Scolopendridae | 5 |
| Insecta | 69 | Coleoptera | 10 | Carabidae^18^ | 1 |
|  |  |  | 10 | Curculionidae^16^ | 1 |
|  |  |  |  | Scarabaeidae | 4 |
|  |  | Diptera^16^ | 2 | Asilidae^16^ | 2 |
|  |  | Hemiptera^16^ | 1 |  |  |
|  |  | Hymenoptera^18^ | 2 | Ichneumonidae^18^ | 2 |
|  |  | Lepidoptera | 3 | Noctuidae^18^ | 2 |
|  |  | Mantodea^18^ | 2 | Mantidae^18^ | 2 |
|  |  | Neuroptera^18^ | 1 |  |  |
|  |  | Orthoptera | 57 | Acrididae^16^ | 2 |
|  |  |  |  | Gryllidae^18^ | 1 |
|  |  |  |  | Rhaphidophoridae | 4 |
|  |  |  |  | Tettigoniidae | 30 |
|  |  | Phasmatodea^18^ | 3 | Lonchodidae^18^ | 3 |
|  |  | Psocoptera^18^ | 1 | Lonchodidae^18^ | 3 |

^16^ Designates taxa found only in *16S* *rRNA* sequence dataset.

^18^ Designates taxa found only in *18S* *rRNA* sequence dataset.

* Strongest DNA sequence match to Family Anostostomatidae, which are not known to occur in Arizona. The phylogenetic sister taxon (Vandergast et al. 2017), Stenopelmatidae, is common Arizona.

Vandergast, A. G., Weissman, D. B., Wood, D. A., Rentz, D. C., Bazelet, C. S., & Ueshima, N. (2017). Tackling an intractable problem: Can greater taxon sampling help resolve relationships within the Stenopelmatoidea (Orthoptera: Ensifera)?. *Zootaxa*, 4291, 1-33.

**Table S3.2.b.** *Leptonycteris yerbabuenae*. Numbers of *L. yerbabuenae* guano samples from Fort Huachuca, AZ, containing *16S* *rRNA* and *18S* *rRNA* amplified sequence variants (ASVs) that were classified to likely prey items. N_C_ = number of guano samples containing ASVs classified to the corresponding class, N_O_ = number of guano samples containing ASVs classified to the corresponding order, N_F_ = number of guano samples containing ASVs classified to the corresponding family, and N_G_ = number of guano samples containing ASVs classified to the corresponding genus.

| **Class** | **N_C_** | **Order** | **N_O_** | **Family** | **N_F_** | **Genus** | **N_G_** |
| --- | --- | --- | --- | --- | --- | --- | --- |
| Arachnida^18^ | 1 | Araneae^18^ | 1 | Salticidae^18^ | 1 |  |  |
| Insecta | 2 | Lepidoptera^16^ | 1 | Noctuidae^16^ | 1 | *Spodoptera*^16^ | 1 |
|  |  | Orthroptera | 5 | Tettigoniidae^16^ | 1 |  |  |
|  |  | Thysanoptera^18^ | 1 | Thripidae^18^ | 1 |  |  |

^16^ Designates taxa found only in *16S* *rRNA* sequence dataset.

^18^ Designates taxa found only in *18S* *rRNA* sequence dataset.

**Table S3.2.c.** *Myotis thysanodes*. Numbers of *M. thysanodes* guano samples from Fort Huachuca, AZ, containing *16S* *rRNA* amplified sequence variants (ASVs) that were classified to likely prey items. N_C_ = number of guano samples containing ASVs classified to the corresponding class, N_O_ = number of guano samples containing ASVs classified to the corresponding order, N_F_ = number of guano samples containing ASVs classified to the corresponding family, and N_G_ = number of guano samples containing ASVs classified to the corresponding genus.

| **Class** | **N_C_** | **Order** | **N_O_** | **Family** | **N_F_** | **Genus** | **N_G_** |
| --- | --- | --- | --- | --- | --- | --- | --- |
| Insecta | 2 | Lepidoptera | 2 | Noctuidae | 2 | *Helicoverpa* | 2 |

**Table S3.2.d.** *Myotis velifer*/*M. yumanensis*. Numbers of *Myotis velifer*/*M. yumanensis* guano samples from Fort Huachuca, AZ, containing *16S* *rRNA* and *18S* *rRNA* amplified sequence variants (ASVs) that were classified to likely prey items. N_C_ = number of guano samples containing ASVs classified to the corresponding class, N_O_ = number of guano samples containing ASVs classified to the corresponding order, N_F_ = number of guano samples containing ASVs classified to the corresponding family, N_G_ = number of guano samples containing ASVs classified to the corresponding genus, and N_S_ = number of guano samples containing ASVs classified to the corresponding species.

| **Class** | **N_C_** | **Order** | **N_O_** | **Family** | **N_F_** | **Genus** | **N_G_** | **Species** | **N_s_** |
| --- | --- | --- | --- | --- | --- | --- | --- | --- | --- |
| Arachnida^18^ | 1 | Araneae^18^ | 1 | Araneidae^18^ | 1 |  |  |  |  |
| Insecta | 46 | Blattodea | 2 | Corydiidae | 1 |  |  |  |  |
|  |  | Coleoptera | 41 | Anobiidae^16^ | 1 | *Tricorynus*^16^ | 1 |  |  |
|  |  |  |  | Elateridae^18^ | 1 | *Anchastus*^18^ |  |  |  |
|  |  |  |  | Scarabaeidae | 36 |  |  |  |  |
|  |  |  |  | Staphylinidae^18^ | 1 |  |  |  |  |
|  |  |  |  | Tenebrionidae^18^ | 7 |  |  |  |  |
|  |  | Diptera^16^ | 1 | Chironomidae^16^ | 1 |  |  |  |  |
|  |  | Hymenoptera^18^ | 3 | Bethylidae^18^ | 2 | *Pseudisobrachium*^18^ | 2 |  |  |
|  |  |  |  | Tiphiidae^18^ | 1 | *Brachycistis*^18^ | 1 |  |  |
|  |  | Lepidoptera^16^ | 5 | Papilionidae^16^ | 1 |  |  |  |  |
|  |  |  |  | Plutellidae^16^ | 1 | *Plutella*^16^ | 1 | *Plutella xylostella*^16^ | 1 |
|  |  |  |  | Tortricidae^16^ | 3 |  |  |  |  |
|  |  | Neuroptera^18^ | 6 | Hemerobiidae^18^ | 2 | *Micromus*^18^ | 2 |  |  |
|  |  |  |  | Mantispidae^18^ | 1 | *Plega*^18^ | 1 |  |  |
|  |  | Psocoptera^18^ | 1 | Psocidae^18^ | 1 |  |  |  |  |

^16^ Designates taxa found only in *16S* *rRNA* sequence dataset.

^18^ Designates taxa found only in *18S* *rRNA* sequence dataset.

**Table S3.3.a.** *Leptonycteris yerbabuenae*. Numbers of *L. yerbabuenae* guano samples from Fort Huachuca, AZ, containing plant barcode ASVs from either the *trnH-psbA* locus or a portion of the *18S* *rRNA* locus. N_C_ = number of guano samples containing ASVs classified to the corresponding class, N_O_ = number of guano samples containing ASVs classified to the corresponding order, N_F_ = number of guano samples containing ASVs classified to the corresponding family, and N_G_ = number of guano samples containing ASVs classified to the corresponding genus.

| **Class** | **N_C_** | **Order** | **N_O_** | **Family** | **N_F_** | **Genus** | **N_G_** |
| --- | --- | --- | --- | --- | --- | --- | --- |
| Magnoliopsida | 17 | Asparagales | 10 | Asparagaceae | 10 |  |  |
|  |  | Asterales | 2 | Asteraceae | 2 |  |  |
|  |  | Commelinales | 1 | Commelinaceae | 1 |  |  |
|  |  | Fabales | 1 | Fabaceae | 1 | *Calliandra* | 1 |
|  |  | Gentianales | 1 | Rubiaceae | 1 |  |  |
|  |  | Lamiales | 1 | Verbenaceae | 1 |  |  |
|  |  | Myrtales | 6 | Myrtaceae | 6 |  |  |
|  |  | Poales | 1 | Poaceae | 1 |  |  |
|  |  | Rosales | 1 | Rhamnaceae | 1 |  |  |

**Table S3.3.b.** *Antrozous pallidus*. Numbers of *A. pallidus* guano samples from Fort Huachuca, AZ, containing plant barcode ASVs from either the *trnH-psbA* locus or a portion of the *18S* *rRNA* locus. N_C_ = number of guano samples containing ASVs classified to the corresponding class, N_O_ = number of guano samples containing ASVs classified to the corresponding order, N_F_ = number of guano samples containing ASVs classified to the corresponding family, and N_G_ = number of guano samples containing ASVs classified to the corresponding genus.

| **Class** | **N_C_** | **Order** | **N_O_** | **Family** | **N_F_** | **Genus** | **N_G_** |
| --- | --- | --- | --- | --- | --- | --- | --- |
| Magnoliopsida | 57 | Asparagales^18^ | 25 | Asparagaceae^18^ | 25 |  |  |
|  |  | Asterales | 24 | Asteraceae | 24 | *Erigeron*^TP^ | 1 |
|  |  | Brassicales^18^ | 1 | Brassicaceae^18^ | 1 |  |  |
|  |  | Caryophyllales | 11 | Chenopodiaceae^TP^ | 2 | *Chenopodium*^TP^ | 2 |
|  |  |  |  | Nyctaginaceae^TP^ | 2 |  |  |
|  |  | Fabales | 12 | Fabaceae | 12 | *Calliandra*^TP^ | 1 |
|  |  |  |  |  |  | *Mimosa*^TP^ | 3 |
|  |  |  |  |  |  | *Prosopisa*^TP^ | 6 |
|  |  | Fagales^TP^ | 1 | Juglandaceae^TP^ | 1 |  |  |
|  |  | Gentianales^TP^ | 1 | Rubiaceae^TP^ | 1 |  |  |
|  |  | Lamiales | 7 | Acanthaceae^TP^ | 1 | *Anisacanthus*^TP^ | 1 |
|  |  | Malpighiales^18^ | 5 |  |  |  |  |
|  |  | Poales^18^ | 18 | Poaceae^18^ | 18 |  |  |
|  |  | Rosales | 2 | Rhamnaceae^18^ | 1 |  |  |
|  |  |  |  | Rosaceae^TP^ | 1 | *Cercocarpus*^TP^ | 1 |
|  |  | Solanales^TP^ | 1 | Convolvulaceae^TP^ | 1 | *Dichondra*^TP^ | 1 |
| Pinopsida^18^ | 3 |  |  |  |  |  |  |
| Polypodiopsida^18^ | 2 |  |  |  |  |  |  |

^TP^ Designates taxa found only in *trnH-psbA* sequence dataset.

^18^ Designates taxa found only in *18S* *rRNA* sequence dataset.

**Table S3.3.c.** *Myotis velifer*/*M. yumanensis*. Numbers of *M. velifer*/*M. yumanensis* guano samples from Fort Huachuca, AZ, containing plant barcode ASVs from either the *trnH-psbA* locus or a portion of the *18S* *rRNA* locus. N_C_ = number of guano samples containing ASVs classified to the corresponding class, N_O_ = number of guano samples containing ASVs classified to the corresponding order, N_F_ = number of guano samples containing ASVs classified to the corresponding family, N_G_ = number of guano samples containing ASVs classified to the corresponding genus.

| **Class** | **N_C_** | **Order** | **N_O_** | **Family** | **N_F_** | **Genus** | **N_G_** |
| --- | --- | --- | --- | --- | --- | --- | --- |
| Magnoliopsida | 23 | Asparagales^18^ | 5 | Asparagaceae^18^ | 5 |  |  |
|  |  | Asterales^TP^ | 1 | Asteraceae^TP^ | 1 | *Heterotheca*^TP^ | 1 |
|  |  | Caryophyllales^TP^ | 2 | Amaranthaceae^TP^ | 1 | *Amaranthus*^TP^ | 1 |
|  |  |  |  | Chenopodiaceae^TP^ | 1 | *Dysphania*^TP^ | 1 |
|  |  | Ericales^TP^ | 12 | Ericaceae^TP^ | 12 | *Arctostaphylos*^TP^ | 12 |
|  |  | Fabales | 15 | Fabaceae | 15 | *Calliandra*^TP^ | 9 |
|  |  |  |  |  |  | *Desmanthus*^TP^ | 3 |
|  |  |  |  |  |  | *Mimosa*^TP^ | 12 |
|  |  |  |  |  |  | *Prosopis*^TP^ | 3 |
|  |  | Fagales^TP^ | 4 | Fagaceae^TP^ | 1 | *Quercus*^TP^ | 1 |
|  |  |  |  | Juglandaceae^TP^ | 1 |  |  |
|  |  | Gentianales^TP^ | 1 | Apocynaceae^TP^ | 1 | *Cynanchum*^TP^ | 1 |
|  |  | Poales^18^ | 7 | Poaceae^18^ | 7 |  |  |
|  |  | Rosales^TP^ | 6 | Rosaceae^TP^ | 6 | *Cercocarpus*^TP^ | 6 |
|  |  | Solanales^TP^ | 1 | Convolvulaceae^TP^ | 1 | *Evolvulus*^TP^ | 1 |
| Pinopsida | 3 | Pinales^TP^ | 1 | Pinaceae^TP^ | 1 | *Pinus*^TP^ | 1 |

^TP^ Designates taxa found only in *trnH-psbA* sequence dataset.

^18^ Designates taxa found only in *18S* *rRNA* sequence dataset.
