# Supplementary material for: Multifaceted DNA metabarcoding of guano to uncover multiple classes of ecological data in two different bat communities
Source: Evol Appl. 2022 Jun 29;15(7):1189–200. doi: 10.1111/eva.13425 (PMC9309442; doi:10.1111/eva.13425)
Supplement: Supplementary file 5 — Table S4 [file EVA-15-1189-s002.docx]

**Table S4.1.a.** *Eptesicus fuscus*. Numbers of *E. fuscus* guano samples from Fort Drum, NY containing *18S* *rRNA* amplified sequence variants (ASVs) that were classified to groups known to contain bat parasites. N_P_ = number of guano samples containing ASVs classified to the corresponding phylum, N_C_ = number of guano samples containing ASVs classified to the corresponding class, and N_O_ = number of guano samples containing ASVs classified to the corresponding order.

| **Phylum** | **N_P_** | **Class** | **N_C_** | **Order** | **N_O_** |
| --- | --- | --- | --- | --- | --- |
| Platyhelminthes | 1 | Trematoda | 1 | Plagiorchiida | 1 |

**Table S4.1.b.** *Myotis lucifugus*. Numbers of *Myotis lucifugus* guano samples from Fort Drum, NY containing *18S* *rRNA* and *16S* *rRNA* amplified sequence variants (ASVs) that were classified to groups known to contain bat parasites. N_P_ = number of guano samples containing ASVs classified to the corresponding phylum, N_C_ = number of guano samples containing ASVs classified to the corresponding class, N_O_ = number of guano samples containing ASVs classified to the corresponding order, N_F_ = number of guano samples containing ASVs classified to the corresponding family, and N_G_ = number of guano samples containing ASVs classified to the corresponding genus.

| **Phylum** | **N_P_** | **Class** | **N_C_** | **Order** | **N_O_** | **Family** | **N_F_** | **Genus** | **N_G_** |
| --- | --- | --- | --- | --- | --- | --- | --- | --- | --- |
| Aplicomplexa | 78 | Conoidasida | 78 | Eucoccidiorida^18^ | 76 | Eimeriidae | 75 | *Eimeria*^16^ | 12 |
|  |  |  |  |  |  |  |  | *Isospora*^16^ | 1 |
|  |  |  |  |  |  | Hepatozoidae^18^ | 3 | *Hepatozoon*^18^ | 3 |
| Arthropoda | 38 | Arachnida | 26 | Mesostigmata^16^ | 2 | Spinturnicidae^16^ | 2 | *Spinturnix*^16^ | 2 |
|  |  |  |  | Sarcoptiformes^18^ | 1 | Rosensteiniidae^18^ | 1 | *Nycteriglyphites*^18^ | 1 |
|  |  |  |  | Trombidiformes | 23 | Cheyletidae^16^ | 18 | *Neochelacheles*^16^ | 18 |
|  |  |  |  |  |  | Demodicidae^16^ | 1 | *Demodex*^16^ | 1 |
|  |  |  |  |  |  | Myobiidae | 4 | *Myobia*^16^ | 1 |
|  |  | Insecta | 12 | Hemiptera^16^ | 8 | Cimicidae^16^ | 8 | *Cimex*^16^ | 8 |
|  |  |  |  | Siphonaptera^18^ | 4 |  |  |  |  |
| Nematoda^18^ | 1 |  |  |  |  |  |  |  |  |
| Platyhelminthes | 27 | Cestoda^18^ | 4 | Cyclophyllidea^18^ | 4 | Hymenolepididae^18^ | 4 | *Diploposthe*^18^ | 2 |
|  |  |  |  |  |  |  |  | *Fimbriaria*^18^ | 2 |
|  |  | Trematoda | 23 | Plagiorchiida | 23 | Lecithodendriidae | 10 |  |  |

^18^Designates taxa found only in *18S* *rRNA* sequence dataset.

^16^ Designates taxa found only in *16S* *rRNA* sequence dataset.

**Table S4.2.a.** *Antrozous pallidus*. Numbers of *Antrozous pallidus* guano samples from Fort Huachuca, AZ containing *18S* *rRNA* amplified sequence variants (ASVs) that were classified to groups known to contain bat parasites. N_P_ = number of guano samples containing ASVs classified to the corresponding phylum, N_C_ = number of guano samples containing ASVs classified to the corresponding class, N_O_ = number of guano samples containing ASVs classified to the corresponding order, N_F_ = number of guano samples containing ASVs classified to the corresponding family, and N_G_ = number of guano samples containing ASVs classified to the corresponding genus.

| **Phylum** | **N_P_** | **Class** | **N_C_** | **Order** | **N_O_** | **Family** | **N_F_** | **Genus** | **N_G_** |
| --- | --- | --- | --- | --- | --- | --- | --- | --- | --- |
| Apicomplexa | 49 | Conoidasida | 49 | Eucoccidiorida | 49 | Eimeriidae | 49 |  |  |
| Arthropoda | 12 | Arachnida | 10 | Mesostigmata | 1 |  |  |  |  |
|  |  |  |  | Sarcoptiformes | 7 | Rosensteiniidae | 7 | *Nycteriglyphites* | 7 |
|  |  |  |  | Trombidiformes | 2 | Myobiidae | 1 |  |  |
|  |  |  |  |  |  | Trombiculidae | 1 |  |  |
|  |  | Insecta | 4 | Siphonaptera | 4 |  |  |  |  |
| Euglenzoa | 9 | Kinetoplastea | 9 | Trypanosomatida | 9 | Trypanosomatidae | 9 |  |  |
| Nematoda | 1 | Chromodorea | 1 | Rhabditida | 1 | Thelaziidae | 1 | *Oxyspirura* | 1 |
| Platyhelminthes | 3 | Cestoda | 3 | Cyclophyllidea | 3 | Hymenolepididae | 3 |  |  |

**Table S4.2.b.** *Leptonycteris yerbabuenae*. Numbers of *Leptonycteris yerbabuenae* guano samples from Fort Huachuca, AZ containing *18S* *rRNA* amplified sequence variants (ASVs) that were classified to groups known to contain bat parasites. N_P_ = number of guano samples containing ASVs classified to the corresponding phylum, N_C_ = number of guano samples containing ASVs classified to the corresponding class, N_O_ = number of guano samples containing ASVs classified to the corresponding order, N_F_ = number of guano samples containing ASVs classified to the corresponding family, and N_G_ = number of guano samples containing ASVs classified to the corresponding genus.

| **Phylum** | **N_P_** | **Class** | **N_C_** | **Order** | **N_O_** | **Family** | **N_F_** | **Genus** | **N_G_** |
| --- | --- | --- | --- | --- | --- | --- | --- | --- | --- |
| Apicomplexa | 12 | Conoidasida | 12 | Eucoccidiorida | 12 | Eimeriidae | 12 |  |  |
| Arthropoda | 3 | Arachnida | 3 | Sarcoptiformes | 3 |  |  |  |  |
|  |  |  |  | Trombidiformes | 1 | Rosensteiniidae | 2 | *Nycteriglyphites* | 2 |
|  |  |  |  |  |  | Myobiidae | 1 |  |  |
| Platyhelminthes | 13 | Cestoda | 13 | Cyclophyllidea | 13 | Hymenolepididae | 13 |  |  |

**Table S4.2.c.** *Myotis thysanodes*. Numbers of *Myotis thysanodes* guano samples from Fort Huachuca, AZ containing *18S* *rRNA* amplified sequence variants (ASVs) that were classified to groups known to contain bat parasites. N_P_ = number of guano samples containing ASVs classified to the corresponding phylum, N_C_ = number of guano samples containing ASVs classified to the corresponding class, N_O_ = number of guano samples containing ASVs classified to the corresponding order, and N_F_ = number of guano samples containing ASVs classified to the corresponding family.

| **Phylum** | **N_P_** | **Class** | **N_C_** | **Order** | **N_O_** | **Family** | **N_F_** |
| --- | --- | --- | --- | --- | --- | --- | --- |
| Apicomplexa | 2 | Conoidasida | 2 | Eucoccidiorida | 2 | Eimeriidae | 2 |
|  |  |  |  |  |  |  |  |

**Table S4.2.d.** *Myotis velifer/M. yumanensis*. Numbers of *Myotis velifer/M. yumanensis* guano samples from Fort Huachuca, AZ containing *18S* *rRNA* amplified sequence variants (ASVs) that were classified to groups known to contain bat parasites. N_P_ = number of guano samples containing ASVs classified to the corresponding phylum, N_C_ = number of guano samples containing ASVs classified to the corresponding class, N_O_ = number of guano samples containing ASVs classified to the corresponding order, N_F_ = number of guano samples containing ASVs classified to the corresponding family, and N_G_ = number of guano samples containing ASVs classified to the corresponding genus.

| **Phylum** | **N_P_** | **Class** | **N_C_** | **Order** | **N_O_** | **Family** | **N_F_** | **Genus** | **N_G_** |
| --- | --- | --- | --- | --- | --- | --- | --- | --- | --- |
| Apicomplexa | 45 | Conoidasida | 45 | Eucoccidiorida | 45 | Cryptosporidiidae | 1 | *Cryptosporidium* | 1 |
|  |  |  |  |  |  | Eimeriidae | 45 |  |  |
| Arthropoda | 2 | Arachnida | 2 | Sarcoptiformes | 1 |  |  |  |  |
|  |  |  |  | Trombidiformes | 1 | Myobiidae | 1 |  |  |
| Platyhelminthes | 2 | Cestoda | 2 | Cyclophyllidea | 2 | Hymenolepididae | 2 |  |  |
